# Deciphering the Metabolic Impact and Clinical Relevance of N‐Glycosylation in Colorectal Cancer through Comprehensive Glycoproteomic Profiling

**DOI:** 10.1002/advs.202415645

**Published:** 2025-04-26

**Authors:** Guobin Liu, Lu Chen, Jingxiang Zhao, Yue Jiang, Yarong Guo, Xiang Mao, Xuelian Ren, Kun Liu, Qi Mei, Qunyi Li, He Huang

**Affiliations:** ^1^ State Key Laboratory of Chemical Biology Shanghai Institute of Materia Medica Chinese Academy of Sciences Shanghai 201203 China; ^2^ Department of Pharmacy Huashan Hospital Fudan University Shanghai 200040 China; ^3^ School of Life Science and Technology ShanghaiTech University Shanghai 201203 China; ^4^ Shandong Laboratory of Yantai Drug Discovery Bohai Rim Advanced Research Institute for Drug Discovery Yantai 264117 China; ^5^ School of Mechanical Engineering and Automation Northeastern University Shenyang 110819 China; ^6^ Department of Digestive System Oncology Shanxi Bethune Hospital Shanxi Academy of Medical Sciences Tongji Shanxi Hospital Third Hospital of Shanxi Medical University Taiyuan 030032 China; ^7^ Department of Surgery Huashan Hospital Fudan University Shanghai 200040 China; ^8^ School of Pharmaceutical Science and Technology Hangzhou Institute for Advanced Study University of Chinese Academy of Sciences Hangzhou 310024 China; ^9^ Department of Oncology Tongji Hospital Tongji Medical College Huazhong University of Science and Technology Wuhan 430030 China

**Keywords:** clinical relevance, colorectal cancer, functions, glycoproteomics, intact *N*‐glycopeptides

## Abstract

Colorectal cancer (CRC) progression is driven by complex metabolic alterations, including aberrant N‐glycosylation patterns that critically influence tumor development. However, the metabolic and functional roles of N‐glycosylation in CRC remain poorly understood. Herein, comprehensive proteomic and N‐linked intact glycoproteomics analyses are performed on 45 CRC tumors, and normal adjacent tissues (NATs) are matched, identifying 7125 intact N‐glycopeptides from 704 glycoproteins. Through analysis of glycoform expression profiles and structural characteristics, a glycosylation site–protein function association network is constructed to uncover metabolic dysregulation driven by N‐glycosylation in CRC. Moreover, an arithmetic model is developed that integrates N‐glycan expression patterns, which effectively distinguishes tumors from NATs, reflecting metabolic reprogramming in cancer. These findings identify Chloride Channel Accessory 1 (CLCA1) and Olfactomedin 4 (OLFM4) as potential metabolic biomarkers for CRC diagnosis. Immunohistochemistry and Cox regression analyses validated the prognostic power of these markers. Notably, the critical role of specific N‐glycosylation at N196 of Adipocyte plasma membrane‐associated protein (APMAP) is highlighted, a key player in tumor metabolism and CRC progression, providing a potential target for therapeutic intervention. These findings offer valuable insights into the metabolic roles of N‐glycosylation in CRC, advancing biomarker discovery, enhancing metabolic‐based diagnostic precision, and improving personalized treatment strategies targeting cancer metabolism.

## Graphical abstract

The comprehensive proteomic and N‐glycoproteomic analyses of 45 colorectal cancer tissues with matched normal adjacent tissues identified 7125 intact N‐glycopeptides from 704 glycoproteins. A glycosylation site‐protein function network revealing metabolic dysregulation is constructed and a model differentiating tumors from normal tissues is developed. CLCA1 and OLFM4 are validated as diagnostic biomarkers, while site‐specific glycosylation at APMAP‐N196 emerged as a therapeutic target.

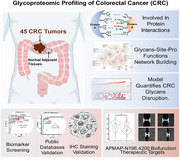

## 1 Introduction

Colorectal cancer (CRC) progression involves multifaceted genetic/epigenetic alterations, posing significant challenges to global health.^[^ ^1^, ^2^ ^]^ While aberrant N‐glycosylation is increasingly recognized as pivotal in CRC pathogenesis,^[^ ^3^, ^4^ ^]^ yet key knowledge gaps persist. First, the biological functions of complete N‐glycan structures and their modification sites remain unelucidated.^[^ ^5^, ^6^, ^7^ ^]^ Second, accurately and comprehensively quantifying the impact of N‐glycosylation on protein state changes continues to be a significant challenge.^[^ ^8^, ^9^, ^10^, ^11^ ^]^ Finally, due to the extensive structural diversity and dynamic nature of N‐glycans, accurately pinpointing their specific functions and regulatory mechanisms has remained challenging, limiting our understanding of N‐glycosylation in cancer.^[^ ^12^ ^]^

Recent advances in N‐glycoproteomics, particularly enrichment techniques, and spectral interpretation algorithms,^[^ ^13^, ^14^ ^]^ now enable precise characterization of CRC pathologically relevant N‐glycosylation. N‐glycosylation not only encodes a wealth of information beyond the primary protein sequence but also exhibits specific site alterations that can significantly impact the efficacy of cancer therapy targets, such as the N‐glycosylation states of Programmed death‐ligand 1 (PD‐L1)/ Programmed Cell Death Protein‐1 (PD‐1) and Epidermal growth factor receptor (EGFR), which are known to affect the therapeutic outcomes of treatments targeting these molecules.^[^ ^15^, ^16^ ^]^ Notably, N‐glycosylation patterns themselves are emerging as CRC diagnostic/prognostic biomarkers, as their disease‐specific alterations often precede morphological change.^[^ ^17^, ^18^, ^19^ ^]^ This analytical power uniquely reveals microenvironmental dysregulation independent of protein abundance/complexity, with particular value for early‐stage biomarker discovery.^[^ ^20^ ^]^

Therefore, to gain a deeper understanding of the relationship between N‐glycosylation dysregulation and CRC progression, and to identify potential therapeutic targets and biomarkers for CRC, we performed comprehensive proteomic and N‐intact glycoproteomics analyses on 45 CRC tumors and their matched normal adjacent tissues (NATs). In total, 7125 intact N‐glycopeptides (IGPs) corresponding to 704 glycoproteins were identified. We systematically investigated the potential functions associated with the cellular localization and expression profiles of different glycoforms. By integrating the structural characteristics and distribution differences of these glycoforms, we constructed comprehensive glycosylation site‐protein function association networks to better understand the dysregulation of N‐glycosylation in CRC. Furthermore, we developed an arithmetic model to integrate and characterize the complex global changes in N‐glycans in CRC through asynchronous overexpression of sialylated and mannosylated glycoforms. Additionally, a logistic algorithm combining 8 glycoproteins with the highest abundant N‐glycans distinguished tumor samples from NATs well, with an area under the receiver operating characteristic curve (ROC AUC) of 0.979. Further analysis using random forest and logistic regression identified Chloride Channel Accessory 1 (CLCA1) and Olfactomedin 4 (OLFM4) as potential CRC biomarkers, achieving an AUC of 1 and 0.969, respectively. Immunohistochemistry (IHC) and multivariate Cox regression analysis confirmed the model's ability to predict patient prognosis, highlighting the potential of glycoproteins as diagnostic features for colorectal cancer. Notably, our study found that the N‐glycosylation at the APMAP‐N196 site is significantly reduced in CRC tissues. Functional assays demonstrated that this reduction promotes CRC progression, highlighting the critical role of adipocyte plasma membrane‐associated protein (APMAP)‐N196 N‐glycosylation as a critical bridge in CRC progression. These findings thus pave the way for significant advancements in cancer diagnosis, prognosis, and personalized therapy.

In summary, our study using N‐linked intact glycoproteomics provides valuable perspectives and tools for elucidating the molecular mechanisms of colorectal cancer, particularly in the areas of cancer diagnosis, prognosis, and personalized therapy. These findings offer a scientific basis for better understanding the regulatory mechanisms and specific functions of N‐glycosylation in CRC and provide new avenues for cancer diagnosis and treatment.

## 2 Results

### 2.1 Glycoproteomic Landscape of CRC

**Figure 1:**
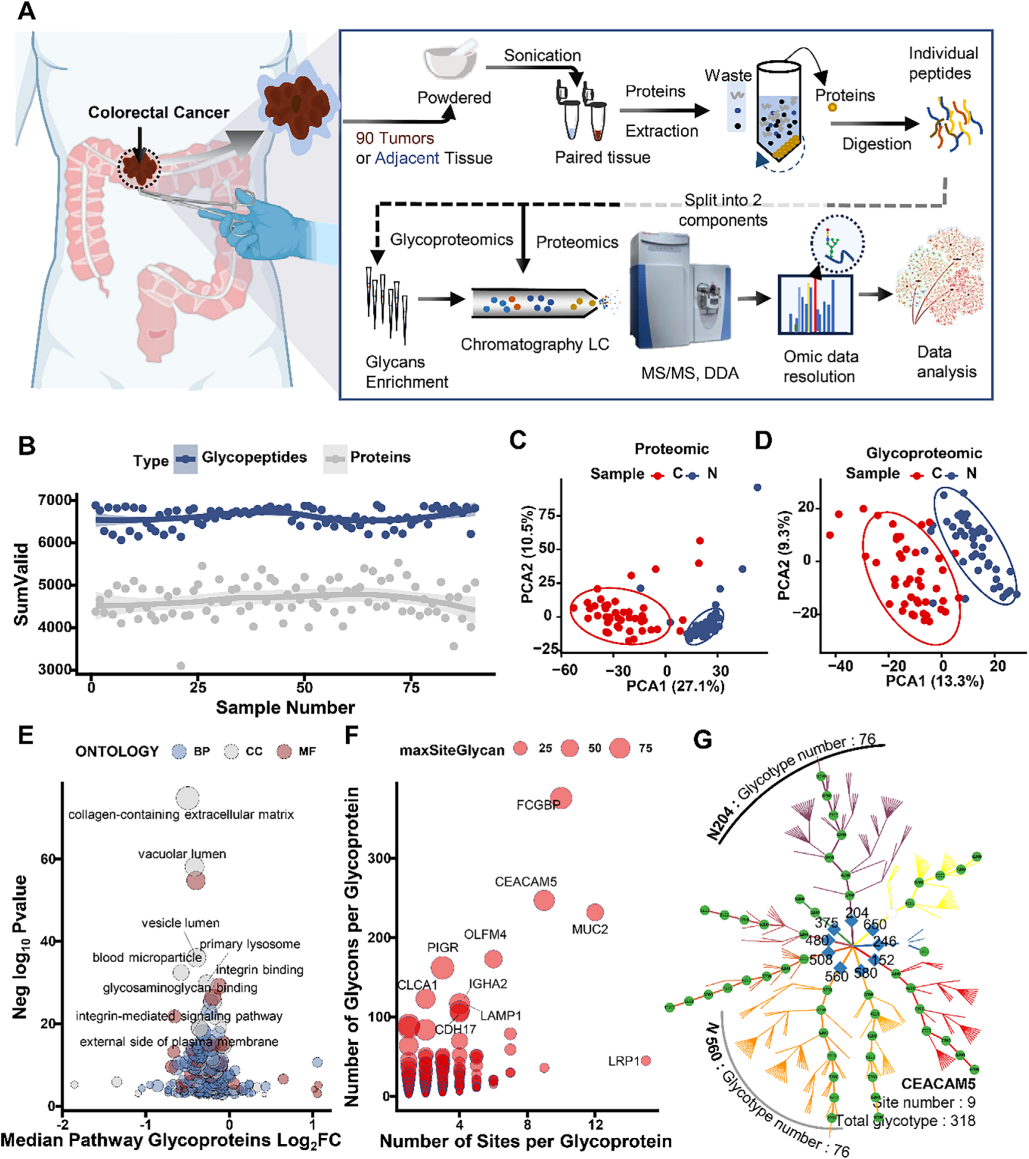
Overview of N‐glycoproteins in the colorectal cancer cohort. (A) Workflow for N‐glycoproteomics sample preparation and subsequent MS analysis. (B) Number of identified glycopeptides (red dots) and proteins (grey dots). (C) Principal component analysis (PCA) of proteomics data. Blue dots represent tumors, red dots represent NATs. (D) PCA of glycoproteomics data. Blue dots represent tumors, red dots represent NATs. (E) GO enrichment analysis of glycoproteins. Differential colors represent differential pathways. (F) Number of glycosites and N‐glycans for each glycoprotein. Differential size indicates the max number of each IGP per N‐glycosites. (G) Glycosites and glycoforms of CEACAM5. Colors represent the N‐glycans of each site, with green indicating 3–11 Hexoses and 2 HexNAc glycans.

To elucidate the role of N‐glycosylation in CRC, we performed in‐depth proteomic and N‐glycoproteomics analyses of 45 collected CRC tumors and NATs (**Figure** **1**A). All tissues were lysed to extract proteins, followed by trypsin digestion. The resulting peptide mixtures were subjected to proteomic analysis or N‐glycosylation analysis after enrichment of IGPs using ZIC‐hydrophilic interaction liquid chromatography (ZIC‐HILIC). The mass spectrometry data were then analyzed for global proteomics and N‐glycoproteomics using MaxQuant and pGlyco3, respectively. Through these processes, we quantified 7567 proteins and 7125 unique IGPs belonging to 704 proteins (Figure 1B, Tables S2 and S3, Supporting Information). Subsequently, we integrated the omics results with the relevant clinical information of the samples (Table S1, Supporting Information). Principal component analysis (PCA) showed that paired NATs effectively clustered together and distinctly separated from tumor samples in both proteomics (Figure 1C) and N‐glycoproteomics (Figure 1D). Compared to proteomics, the N‐glycoproteomics profiles exhibited greater variability among samples (Figure 1C,D and Figure S1A,B, Supporting Information), indicating higher heterogeneity and offering more refined potential avenues for precision molecular therapy. Additionally, Pearson statistical methods were used to compare the correlation between our omics data and public data, including The Cancer Genome Atlas (TCGA) transcriptomic and Clinical Proteomic Tumor Analysis Consortium (CPTAC) proteomic data (Figure S1C–H, Supporting Information). The results supported a positive correlation between transcriptomics and proteomics while showing a weak negative correlation between N‐glycoproteomics and other omics.

To further investigate the subcellular compartments or macromolecular complexes where the detected glycoproteins in our study may function, we performed a Gene Ontology Cellular Component (GO CC) analysis. The results revealed that collagen‐containing extracellular matrix (ECM) (*adjust p* = 1.47E‐67), vacuolar lumen (*adjust p* = 5.99E‐29), and primary lysosome (*adjust p* = 9.86E‐24) were most significantly enriched (Figure 1E). Previous reports have indicated that dysregulation of extracellular proteins is closely associated with the occurrence and progression of colorectal cancer,^[^ ^21^, ^22^ ^]^ and in‐depth studies of ECM protein composition and structure provide new insights for cancer treatment.^[^ ^23^ ^]^ However, most of these ECM proteins have multiple N‐linked glycosylation sites and complex N‐glycans, which greatly complicates the study of N‐glycosylation in tumorigenesis. In our data, 57.4% of N‐linked glycosylation sites were found to possess multiple N‐glycan structures, and 30.7% of glycoproteins had multiple glycosylation sites (Figure 1F and Figure S1I–K, Supporting Information). For example, Carcinoembryonic antigen‐related Cell Adhesion Molecule 5 (CEACAM5), a known prognostic marker for CRC,^[^ ^24^ ^]^ contains up to 319 N‐glycosylation forms with 9 N‐linked glycosylation sites and 99 N‐glycans in our N‐glycoproteomics data (Figure 1G). Overall, N‐glycoproteomics significantly expands the diversity and quantity of membrane and secreted proteins in the proteome, making them potential sources for enhanced tumorigenic capacity and providing numerous targets for cancer treatment.

### 2.2 A Potential Interaction Mode Relies on Different Glycoforms

**Figure 2:**
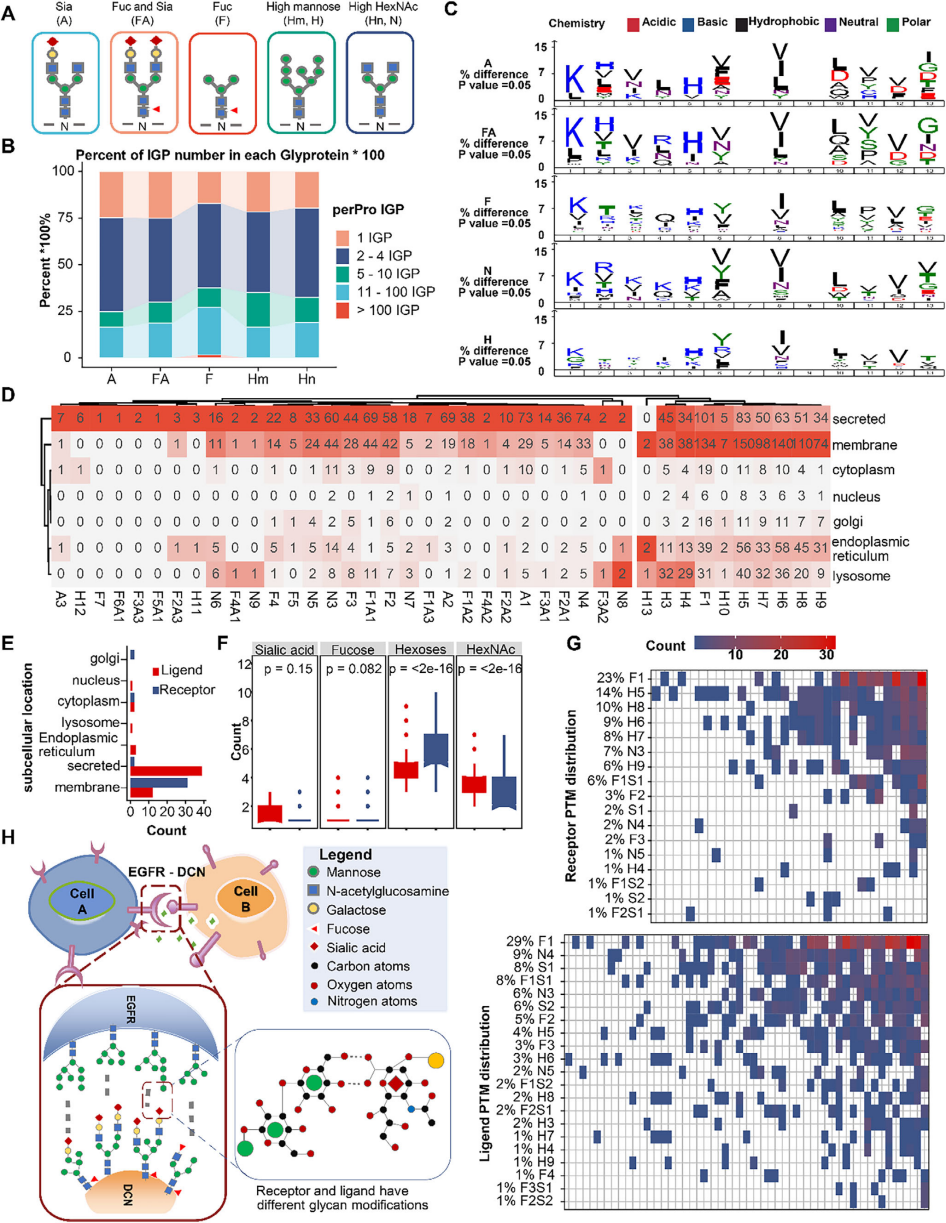
Annotation and analysis of differentially intact glycopeptides cluster. (A) Definition of five glycoforms based on the monosaccharide composition of N‐glycans. S: Sialylated IGP, F: fucosylated IGP, FA: sialylated and fucosylated IGP, H: high mannose IGP, N: high HexNAc IGP. (B) Proportions of singly and multiply N‐glycosylated proteins, colors indicating the number of each IGP per protein. (C) Motif composition of each glycoform. Differential colors represent differential classifications. (D) Summary of UniProt database localization categories for each glycoform. Red saturation including the number of N‐glycan. (E) Bar plot showing the UniProt localization categories for receptor (Blue) and ligand (Red) proteins detected in our data. (F) Number of each monosaccharide composition in receptor (Blue) and ligand (Red) proteins. (G) Heatmap displaying the number of each glycoform per glycoproteins. Each column represents a glycoprotein, with colors indicating glycoforms counts. Row names indicate each glycoform's name and their proportions in receptor (top) and ligand (button). (H) A potential mutual attract/binding mode based on different glycoforms. Ligands are represented in grey color. Mannose, N‐acetylglucosamine, galactose, fucose, and sialic acid are represented by green circles, blue squares, yellow circles, red triangles, and red diamonds, respectively. Carbon, nitrogen, and oxygen atoms are represented by black circles, blue circles, and red circles, respectively.

To explore the impact of the complex structure of N‐glycosylation on its functions, we defined five glycoforms based on monosaccharide composition: sialylated (Sia, A), fucosylated (Fuc, F), sialylated‐fucosylated (FA), high mannose (Hm, H), and high HexNAc (Hn, N) (**Figure** **2**A). By grouping glycoforms according to the presence or absence of sialic acid, fucose, or extended mannose residues, we simplified the complex N‐glycan landscape. N‐glycoproteomics analysis showed different proportions of N‐glycoforms, with Hm (30%) and Fuc (38%) forms dominating in CRC N‐glycoproteomics (Figure S2A, Supporting Information). The Hm group contained the largest number of glycoproteins (Figure S2A, Supporting Information), while N‐glycosylation sites in the Fuc group were more clustered on the same proteins (Figure 2B). Most observed non‐Hm glycoforms were associated with lower mannose content (Figure S2B, Supporting Information), representing a low N‐glycan skeleton, particularly for Fuc. Fucosylation refers to the terminal modification of the peptide proximal GlcNAc moiety within the Hn pentasaccharide core,^[^ ^20^ ^]^ and IGPs were largely restricted to Asn‐X‐Ser/Thr/Cys (X not equal to Pro) consensus sequences^[^ ^25^ ^]^ (Figure S2C, Supporting Information). Generally, human N‐glycosylation pathways involve at least 173 glycosyltransferases,^[^ ^26^ ^]^ with their alternative expression contributing to the generation of diverse glycoforms. To investigate whether there are differences among various glycoform IGP motifs, we compared the amino acids surrounding the consensus sequences in the human proteome and found a notable preference for acidic amino acids (e.g., Asp and Glu) in Sia (A, +4, +6, +7, −1, −5) and FA (+4, +6, +7), while polar amino acids such as Tyr or Thr tended to be located in Hm and Fuc (−1, −5) (Figure 2C). For Hn, positively charged amino acids are generally found at positions (−2, −3, −4, −5), and hydrophobic amino acids mainly at positions (−1, +1, +3, +4, +5).

By analyzing the subcellular localization preferences of different glycoforms, we found that both Sia and high Hn glycoproteins are predominantly located in secreted and membrane regions but are less frequently found in the endoplasmic reticulum (ER) compared to Hm (Figure S2D–F, Supporting Information). Additionally, we further annotated IGPs based on the degree of monosaccharide composition (Figure S3A, Supporting Information). UniProt subcellular localization annotations revealed a similar distribution pattern for Hm glycoproteins in membranes and ER, while Sia glycoproteins were mainly targeted as secreted proteins, and F1‐glycoproteins (mainly Fuc) exhibited the same localization preference as Hm glycoproteins (Figure 2B).

The binding of secreted ligands to specific cell surface receptors is crucial for coordinating various biological processes such as proliferation, migration, and differentiation.^[^ ^27^, ^28^, ^29^ ^]^ Previous studies have demonstrated the modification preferences of certain oligosaccharides in these proteins,^[^ ^30^ ^]^ but the exact mechanisms behind this connection have not been systematically understood due to technological challenges. To depict the relationship between subcellular localization and different N‐glycans, primarily Sia and Hm, we specifically extracted proteins annotated as receptors and ligands from the celltalker package^[^ ^31^ ^]^ (Figure S3B, Supporting Information). Localization analysis indicated that receptors were mainly distributed as membrane proteins, while ligands were largely classified as secreted and membrane proteins (Figure 2E). In terms of N‐glycan composition, receptors exhibited a higher proportion of HexNAc compared to ligands, whereas sialic acid was present in a lower proportion, suggesting a higher preference for Hm in receptors (Figure 2F).

Regarding glycoforms, receptors contained an abundance of high Hm, especially structures from H5 to H9, and less Sia compared to ligands (Figure 2G). Notably, sialic acid is a negatively charged carboxylate ester, often located at the terminal of N‐glycosylation, while Hm has multiple hydroxyl groups at the terminal, which may determine specific functions in ligand‐receptor interactions. For example, EGFR, a crucial receptor protein in stimulating both differentiation and proliferation of cancer cells, can be activated by its respective ligands,^[^ ^32^, ^33^ ^]^ such as EGF and Decorin (DCN).^[^ ^34^ ^]^ Notably, although EGFR exhibits seven glycopeptide variants, they all fall within a narrow range of Hm numbers, spanning from H5 to H8. In contrast, DCN presents a more diverse glycosylation profile, characterized by 16 different Sia glycoforms and 10 Hm glycoforms. Among these numerous glycoforms, DCN has only a single instance where the hexoses exceed six. This comparison highlights the distinct glycosylation patterns between receptor EGFR and its ligand DCN, which may have implications for their functional interactions and roles in cancer biology.

By analyzing the correlation between different glycoforms of ligands and receptors, as well as the properties of hydroxyl and carboxyl groups, we proposed a model defining ligand‐receptor connections based on glycoform recognition and mutual attraction (Figure 2H). This model suggests that surface‐modified glycoforms in ligands contain more carboxyl groups, such as sialic acid, while receptors are more likely modified by hydroxyl groups, such as mannose, resulting in differential electrostatic interactions at specific surface sites of proteins,^[^ ^35^, ^36^ ^]^ thereby generating ionic forces that attract and “recognize” each other.^[^ ^7^ ^]^

### 2.3 Impact of N‐Glycosylation Dynamics on Glycoprotein Function and CRC Progression

**Figure 3:**
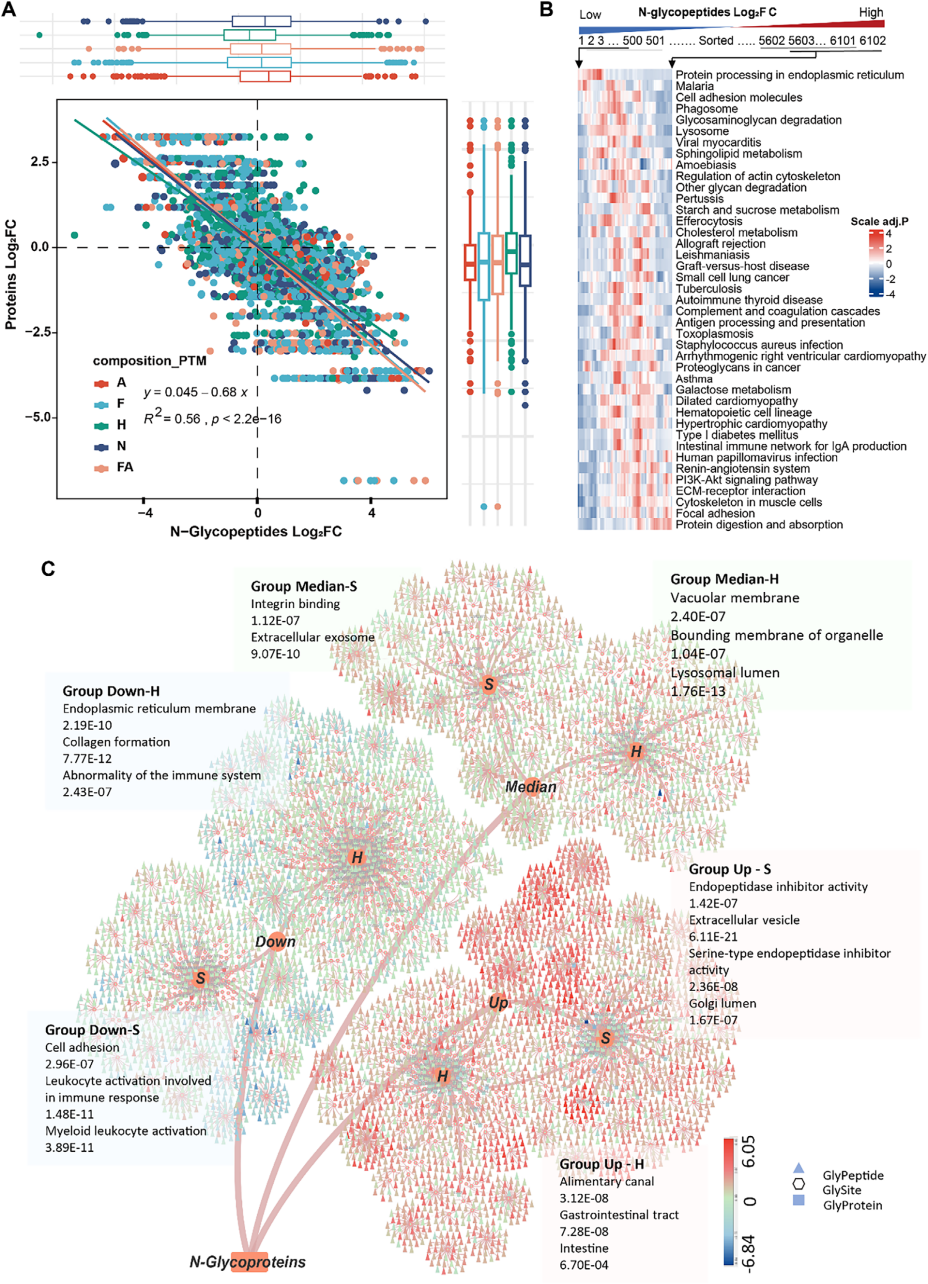
Classification of glycoproteins based on IGP expression patterns and composition. (A) Comparative analysis of differential abundance changes of IGPs and their corresponding proteins in tumors versus NATs. Dot colors represent different glycoforms. (B) Heatmap showing KEGG pathway enrichment of glycoproteins based on IGPs abundance changes in tumors versus NATs. IGPs are sorted by differential abundance changes, and their 500 corresponding proteins were sequentially selected for KEGG pathway enrichment. (C) CRC‐glycoproteins‐glycosites‐glycan tree. Glycoproteins are classified into six groups based on whether two‐thirds of IGP changes are greater than zero and the most common glycoform. Pathways with the highest significance are labeled. Node colors indicate differential changes in IGPs.

Advancements in mass spectrometry and data interpretation techniques have enabled us to quantify the correlations between various IGP dysregulations and functional disturbances during CRC tumor progression. First, we performed a quantitative comparison of the global N‐glycoproteome between tumors and NATs and observed a general upregulation of IGPs in tumors, whereas proteomics showed no significant changes (Figure S4A,B, Supporting Information). Notably, global IGPs were significantly negatively correlated with the expression levels of these glycoproteins (*R* ^2^ = 0.56, p = 2.2E‐16) (**Figures** **3**A and S4C, Supporting Information). Compared to NATs, upregulated glycoproteins in tumors showed significant enrichment in Protein digestion and absorption (*adjust p* < 6.6E‐215), ECM‐receptor interactions (*adjust p* < 6.6E‐215), focal adhesion (*adjust p* < 6.6E‐215), and PI3K‐Akt signaling pathways (*adjust p* < 6.6E‐215), while downregulated glycoproteins were significantly enriched in protein processing in the endoplasmic reticulum (*adjust p* < 2.3E‐321) (Figure 3B).

Furthermore, by comparing the differential abundance of IGPs and glycoprotein levels, we observed distinct expression profiles of various glycoforms between CRC tumors and NATs (Figure 3A). The results indicated that while global IGP glycoforms were higher in tumors than in NATs, their extent of variation was quite different, with the following order: Sia > Fuc (FA) > Hm (Figure S4D, Supporting Information). However, in proteomics, only the levels of Hm‐glycoproteins were significantly higher than non‐Hm‐glycoproteins (Figure S4E, Supporting Information). Considering the associations of Sia with membrane proteins and Hm with secreted proteins, we hypothesized that dynamic changes in glycoforms might lead to protein dysfunction. Our analysis revealed that secretory proteins, particularly those modified with Sia, exhibited more pronounced upregulation (Figure S4F, Supporting Information). In contrast, membrane proteins underwent significant changes in glycoforms, with both upregulation and downregulation occurring in similar proportions, especially for Hm. Secretory proteins modified by upregulated Sia and Hn were found to be more associated with complement (*adjust p* = 2.68E‐15) and coagulation cascades (*adjust p* = 2.52E‐08), while membrane proteins modified by Hm were more associated with cell adhesion molecules (*adjust p* = 1.48E‐03). In conclusion, these results highlight the critical roles of Hm‐ and predominantly Sia‐dominant non‐Hm‐glycoproteins (represented by S‐glycoforms) in CRC progression, which may dictate their differential cellular functions.

Next, to comprehensively characterize the structural and functional differences of various glycoforms, we divided all glycoproteins into six groups based on the median IGP ratios of tumors to NATs and the amounts of Hm‐ and S‐glycoforms, followed by STRING (Search Tool for the Retrieval of Interacting Genes/Proteins) database enrichment analysis (Figure 3C and Figure S4G, Supporting Information). The functional clustering of Up‐S and Down‐Hm groups differed more significantly from those of the other groups (Figure S4G, Supporting Information). The Up‐S clusters showed more enrichment of endopeptidase inhibitor activity (*adjust p* = 1.42E‐07) and were predominantly located in the Golgi lumen (*adjust p* = 1.67E‐07) (Figure 3C). However, the Down‐S group was significantly enriched in cell adhesion (*adjust p* = 2.96E‐07) and immune response pathways (*adjust p* = 1.48E‐11). These results collectively suggest that Sia is highly expressed in Golgi lumen‐resident proteins, contributing to the inhibition of protease activity, while reducing modification in cell adhesion and immune response proteins. Notably, we also identified several gastrointestinal‐related proteins that tended to be downregulated in the Up‐Hm group (Figure 3C), indicating a tissue‐specific nature of glycosylation modifications.

### 2.4 Modeling N‐Glycosylation Variability to Quantify CRC Progression

As mentioned above, protein N‐glycosylation is extensively involved in the progression of CRC through its structural diversity and dynamic changes. Therefore, developing a metric to quantify the extent of glycosylation disruption in CRC patients is crucial for deepening our understanding of tumorigenesis and accelerating cancer drug discovery. However, the complexity and variability of glycosylation pose significant challenges in defining such a metric.

**Figure 4:**
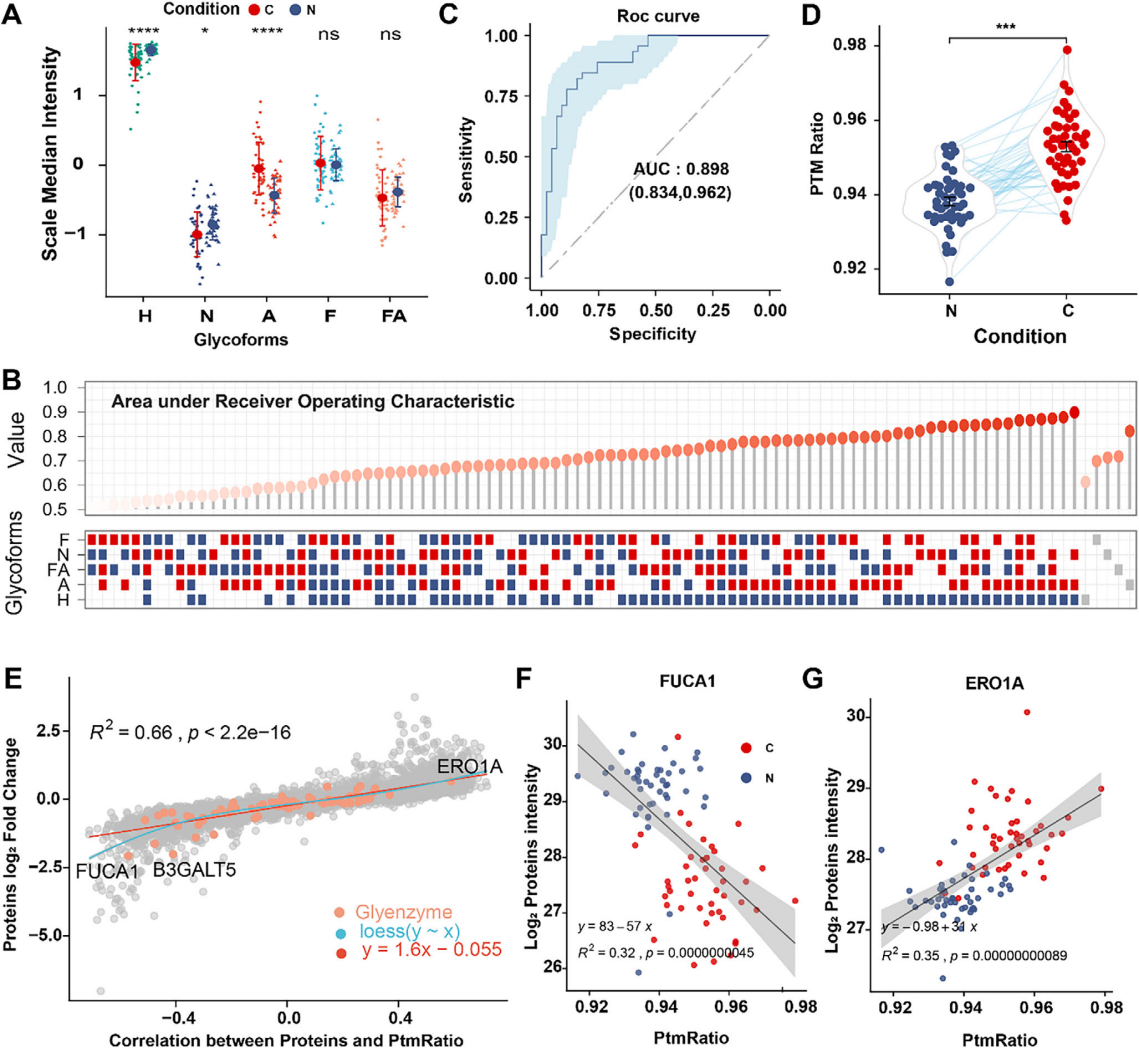
Identification of robust glycoform scores associated with CRC development. (A) Abundance of each glycoforms scaled by P‐value from Wilcoxon rank‐sum test. The point represents the median abundance of glycoforms. Red error bar indicates tumors, and blue indicates NATs. (B) Receiver Operating Characteristic curves (ROC) for median glycoforms abundance and various ratio combinations. The ratio is derived from dividing the red sum (CRC) by the blue sum (NATs). The top panel displays ROC results sorted by the ratios, with red color transparency inversely related to the ROC value. The bottom panel shows the composition of this ratio, with red dots representing the numerator and blue dots representing the denominator. (C) ROC curves for the optimal combination, using median H as the numerator and the sum of A and N as the denominator. (D) Optimal combination results distinguishing tumors (Red dots) from NATs (Blue dots). (E) Correlation between correlation values and log_2_ median differential abundance changes of proteins. Correlation values are derived from Pearson correlation between the optimal combination results and each protein detected in our proteomics analysis (P‐value from Pearson correlation). Orange color represents N‐glycosylation enzymes. (F) Pearson correlation between optimal combination results and FUCA1 abundance. Red dots indicate tumors, and blue indicates NATs. (G) Pearson correlation between optimal combination results and ERO1A abundance. Red dots indicate tumors, and blue indicates NATs.

We accurately scaled the expression of IGPs in all samples to compare the proportions of different glycoforms. Although almost all glycoforms increased in cancer tissues, the alteration rates were clearly asynchronous (Figure 3A and Figure S4D, Supporting Information), with some glycoforms showing significantly lower proportions in tumors compared to NATs (**Figure** **4**A). To accurately link glycoform proportions to CRC occurrence and development, we used the median total abundance of each glycoform per sample as its representative value for comparing differences and assessing glycosylation pattern changes. (Figure 4A). The AUC was used to evaluate the diagnostic potential of the scoring system in CRC patients. We identified Sia as the best marker to distinguish between tumor and normal tissue (AUC = 0.821) (Figure 4B). Second, to assess the synergistic effects across all glycoforms, we compared the median expression of different glycoforms and found that the combined median of Sia and Hm was more effective at distinguishing CRC from NATs (AUC = 0.898) (Figure 4B,C). Notably, the PTM ratio was significantly elevated in tumors compared to adjacent paired tissues (Figure 4D), suggesting that this ratio might be related to the patient rather than the development of the tumor itself (Figure S5A, Supporting Information).

Next, considering the role of glycosylation in protein stability regulation,^[^ ^26^ ^]^ we sought to investigate the correlation between IGPs changes and proteomics dynamics. We found that the overall protein expression was significantly positively correlated with the defined scores (*R* = 0.787, *p* < 2.2e‐16) (Figure 4E), and the proteins with greater variation in CRC were more highly correlated with the score while calculating the denominator or numerator separately did not yield a better Pearson coefficient (Figure S5B,C, Supporting Information). On the other hand, since glycosylation is an enzyme‐catalyzed process, we further explored which enzymes were closely related to the robust scores. We observed that the ratio scores were negatively correlated with Tissue alpha‐L‐fucosidase (FUCA1) and positively correlated with ERO1‐like protein alpha (ERO1A) (Figure 4F,G). Interestingly, FUCA1 catalyzes the hydrolytic cleavage of the terminal fucose residue from IGPs,^[^ ^37^ ^]^ which may explain why FUCA1 was no longer the most relevant factor when calculating the correlation between the numerator and denominator separately with proteomics. These findings demonstrate that global glycosylation profoundly interferes with proteome dysregulation in CRC, and the ratio method used in this study may be more closely associated with the extent of N‐glycosylation disruption in CRC progression.

### 2.5 Identifying Prognostic Signature for Colorectal Cancer Based on Deep Learning

Protein biomarkers facilitate early diagnosis, prognosis estimation, personalized treatment selection, monitoring of treatment response, and prediction of drug resistance, thereby enhancing both diagnostic and therapeutic precision and effectiveness. Emerging evidence also suggests that large‐scale characterization of aberrant glycoproteins has significant potential for the discovery of novel biomarkers and therapeutic targets.^[^ ^17^, ^38^ ^]^

**Figure 5:**
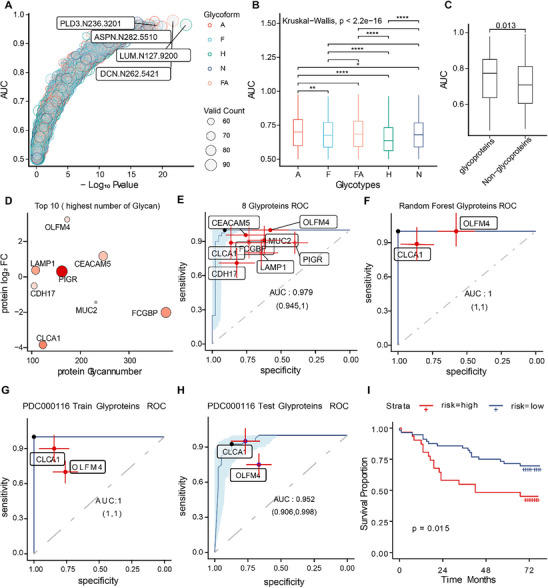
Screening for Potential CRC Prognostic Signature. (A) Point plot showing the AUC values for each IGP. Differential colors represent differential N‐Glycoform. Differential size represents the number of values detected in 90 samples. (B) Boxplot illustrating the AUC for each glycoform. Differential colors represent differential N‐Glycoform. (C) Comparison of AUC values for glycoproteins versus non‐glycoproteins. (D) Top 10 glycoproteins with the highest number of IGPs. The color represents the average number of N‐glycan across all sites in each protein, with higher saturation representing a higher average. (E) ROC curve displaying the performance of multiple glycoproteins logistic regression modules in identifying CRC. Each red dot represents an independent ROC value for each protein. (F) ROC curve showing the results of constructing a random forest model using a combination of CLCA1 and OLFM4. Each red dot represents an independent ROC value for each protein. (G) ROC curve presenting the training results of a random forest model for CLCA1 and OLFM4 using public data. Each red dot represents an independent ROC value for each protein. (H) ROC curve illustrating the testing results of a random forest model for CLCA1 and OLFM4 using public data. Each red dot represents an independent ROC value for each protein. (I) Multivariate survival analyses of IHC patients involved Cox regression analysis. The red line segments represent high risk and the blue color represents low risk.

To further integrate our findings with clinical practice, we explored the potential of N‐glycosylation diagnosis signatures for CRC patients. Our analysis of glycoproteomics data using ROC curves revealed that multiple IGPs could significantly distinguish tumors from NAT (**Figure** **5**A). The discriminative ability varies among different glycoforms, with Sia showing the best performance (Figure 5B). Indeed, glycoproteins were more effective in distinguishing tumors from NATs than non‐glycoproteins (Figure 5C), similar to findings with secreted proteins (Figure S6A–C, Supporting Information).

To expedite the potential clinical application of our findings, we selected the top 10 glycoproteins with the highest abundant N‐glycan modification. Of these, 8 were detected in over 90% of the samples (Figure 5D and Figure S6D, Supporting Information). Using a logistic algorithm, we found that combining these 8 glycoproteins could highly distinguish tumor samples from NATs, achieving an AUC of 0.979 (Figure 5E). Additionally, by combining logistic regression with random forest analysis, we identified two glycoproteins, CLCA1 and OLFM4, as having potential as CRC glycoprotein signatures (Figure 5F). Multi‐factor identification models and joint analysis of these two glycoproteins improved performance in distinguishing tumor tissue from NATs, with AUC values of 1 (Figure 5F) and 0.969 (Figure S6E, Supporting Information) for the random forest and logistic regression algorithms, respectively. This result was further validated with public data, showing an AUC of 1 for the training set (Figure 5G) and 0.952 for the validation set (Figure 4H).

To understand the potential clinical application of our multi‐factorial identification model, we performed an Immunohistochemistry (IHC) analysis and built a multivariate Cox regression proportional hazards model using tissue microarray data including both tumor tissue and NATs (Table S4, Supporting Information). The results confirmed that the model effectively predicts patient prognosis (Figure 5I). Overall, our deep‐learning analysis of glycoproteins in CRC identified several promising modification sites and selected glycoproteins as combined biomarkers. These findings suggest that glycoprotein and glycosylation modification sites, thoroughly analyzed through machine learning, could serve as valuable biomarkers of colorectal cancer.

### 2.6 N‐Glycosylation at APMAP‐N196 was Validated In Vitro as a Potential Regulator of CRC

N‐glycosylation can significantly impact protein interactions, immune recognition, degradation, and signal transduction by altering protein stability, activity, and subcellular localization, thereby affecting its physiological functions.^[^ ^26^ ^]^ Therefore, understanding the role of glycosylation at specific sites is crucial for elucidating protein functions and developing therapeutic strategies.

**Figure 6:**
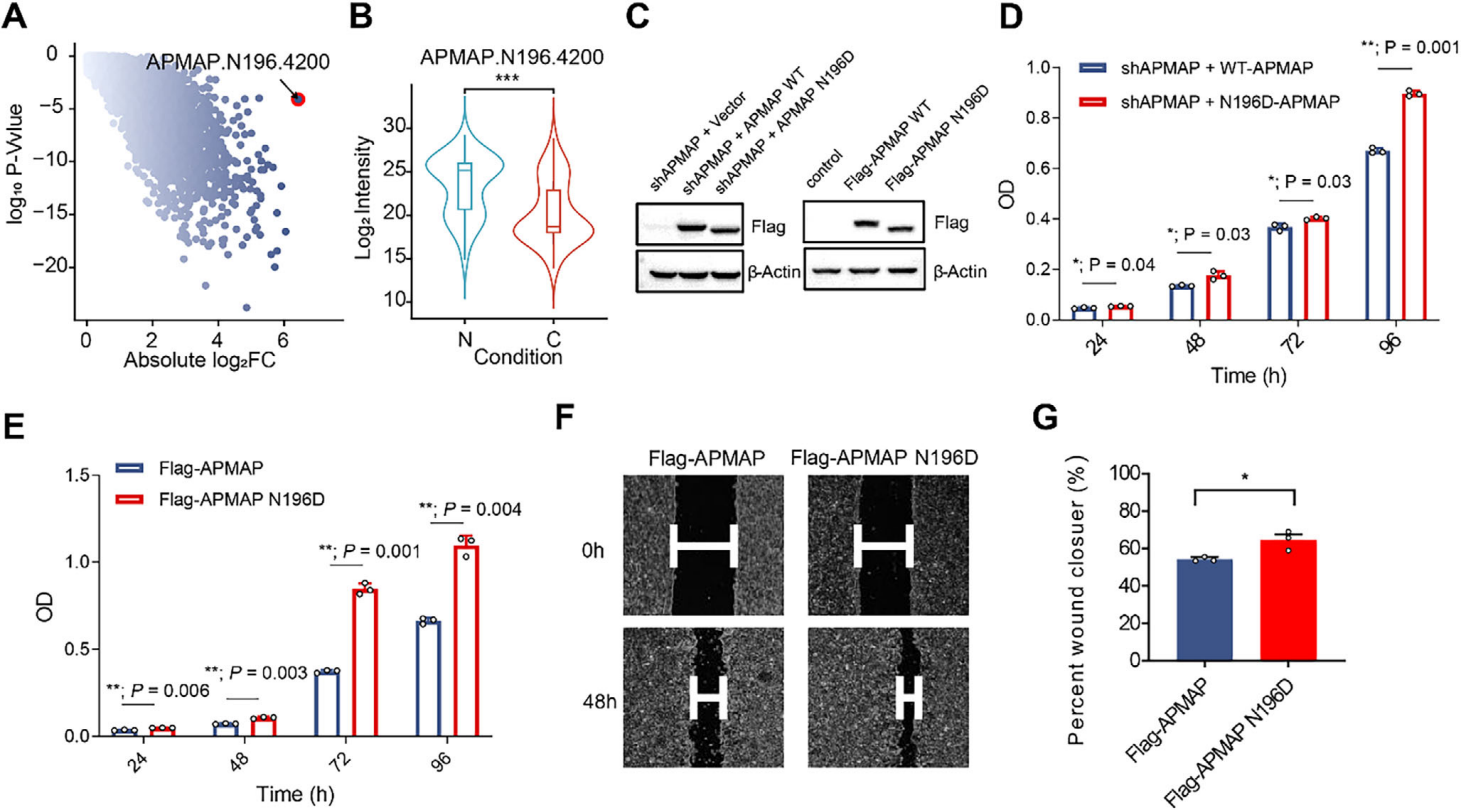
APMAP‐N196 glycosylation contributes to CRC malignancy. (A) Point plot depicting the absolute Log_2_ fold change of IGPs in CRC compared to NATs. (B) Boxplot comparing APMAP.N196.4200 IGP abundance between tumor and NATs. Red color indicates tumors, and blue indicates NATs. (C) Western blot analysis confirming the rescue expression of APMAP in HCT116 cells. (D) Cell proliferation in HCT116 cells, with endogenous APMAP depleted and rescued with either shRNA‐resistant WT or N196D mutant APMAP, was evaluated using a Cell Counting Kit‐8 (CCK‐8) assay. *p < 0.05, ** *p <* 0.01. Differential colors represent differential conditions. (E) CCK‐8 assay assessing the proliferation of HCT116 cells with ectopic overexpression of either WT APMAP or the APMAP‐N196D mutant. *p < 0.05, ** *p <* 0.01. Differential colors represent differential conditions. (F) Representative images from the wound healing assay demonstrating that the APMAP‐N196D mutation promoted cell migration compared with WT. (G) Quantification of HCT116 cell migration levels from the wound healing assay using ImageJ software. *p < 0.05.Differential colors represent differential conditions.

APMAP is a serum glycoprotein implicated in epithelial‐mesenchymal transition (EMT), which promotes tumor invasion and metastasis,^[^ ^39^ ^]^ and is considered a potential biomarker for the early diagnosis of CRC.^[^ ^18^ ^]^ Our analysis reveals that the N‐glycan Hex[4]HexNAc[2]NeuAc[0]Fuc[0] at the N196 site of APMAP (APMAP.N196.4200) is the most significantly altered N‐glycosylation in CRC tissue (**Figure** **6**A). Its IGP abundance in tumors is reduced to just 1.1% of that in NATs (*p* = 8E‐05) (Figure 6B).

To investigate the regulatory role of APMAP‐N196 glycosylation in CRC progression, we generated HCT116 cells with endogenous APMAP depletion and reintroduced either wild‐type (WT) APMAP or the N196D mutant APMAP (Figure 6C). Given APMAP's role in proliferation and migration, we performed CCK‐8 and wound healing assays on HCT116 cells expressing WT or N196D mutant APMAP to assess the impact of N196 glycosylation (Figure 6D–G). Proliferation assays demonstrated that the N196D mutant enhanced proliferation when compared with WT (Figure 6D). Similarly, ectopic overexpression of the APMAP‐N196D led to greater proliferation than WT in HCT116 cells (Figure 6E). Wound healing assay also showed that the N196D mutation increased cell migration relative to WT (Figure 6F,G). These findings indicate that N‐glycosylation at APMAP‐N196 is significantly reduced in tumor tissues compared to NATs, and this reduction further promotes CRC progression, thereby providing new insights into CRC advancement and potential therapeutic interventions.

## 3 Conclusion

The dynamic nature of protein N‐glycosylation and the diversity of N‐glycan structures provide extensive and diverse expansion to the proteome.^[^ ^26^, ^40^ ^]^ However, this complexity also poses significant challenges for the systematic detection and analysis of N‐glycosylation, which has long hindered our understanding of its role in tumors, including CRC.^[^ ^41^, ^42^ ^]^ Much of our current knowledge about CRC relies on insights from other omics approaches. In this study, we present a comprehensive glycoproteomics map of 45 CRC tumors and their paired NATs, identifying 7125 N‐linked IGP corresponding to 704 glycoproteins. Our data visually demonstrated both macro‐ and micro‐heterogeneity of glycosylation, indicating that each glycoprotein can have multiple glycosylation, each potentially modified with different N‐glycans.

Quantitative analysis of these N‐glycosylation sites revealed that most IGPs are membrane‐bound or secreted proteins, some of which, such as EGFR, Mucin‐2 (MUC2), and Carcinoembryonic antigen‐related cell adhesion molecule 5 (CEACAM5), are established cancer biomarkers in immunotherapy.^[^ ^24^, ^43^, ^44^ ^]^ Our analysis particularly highlights the disparity between sialylated and high mannosylated N‐glycans in CRC‐associated glycoproteins. To simplify the heterogeneity of N‐glycans in data analysis, we examined four glycoforms according to the monosaccharide composition. We observed a high degree of sialylation in secreted proteins, suggesting a critical role in extracellular interactions and signal transduction, while proteins enriched in high mannose were primarily associated with membrane‐bound proteins, indicating their potential involvement in cellular recognition or adhesion processes. Glycoproteins in secreted and cell‐surface proteins are crucial for intercellular communication in both host‐host and host‐pathogen interactions.^[^ ^30^ ^]^ Interestingly, the binding affinity of mannose receptors to mannosylated ligands was reduced due to a lack of terminal sialylation,^[^ ^30^, ^45^ ^]^ although the precise mechanism underlying this relationship remains unclear.

Our findings showed that while all assessed glycoforms showed an upregulated trend in CRC, the most notable changes occurred in sialylated and high mannosylated N‐glycans. Based on these observations, we proposed an N‐glycan‐based model emphasizing the balance between mannosylation and sialylation in signal transduction. In addition, we constructed a dynamic network of N‐glycan site‐protein‐signaling pathways, classifying glycoproteins into six groups with distinct cellular functions, based on the numbers of sialylated and high mannosylated N‐glycans. Notably, N‐glycoproteins with reduced sialylation were more closely associated with immune response compared to those with increased sialylation. These findings provide a rationale for developing therapeutic approaches targeting overexpressed glycosylation, especially N‐sialylation in CRC.^[^ ^46^ ^]^

While the N‐glycoproteomic map reveals the complexity of glycosylation in CRC, key questions remain: Which specific glycose alterations are most intimately linked to the etiology and progression of CRC? Could an effective biomarker definitively indicate the relationship between N‐glycan modifications and CRC? To address these, we employed ROC curve analysis, revealing a central role for sialylation in this interplay. N‐glycans synthesis is a multi‐compartmental process involving over 700 glycosyltransferases and glycosidases.^[^ ^47^ ^]^ Our investigation identified aberrant levels of Tissue alpha‐L‐fucosidase (FUCA1) and ERO1‐like protein alpha (ERO1A), which showed the strongest correlations with dysregulated glycosylation scores in CRC, suggesting these enzymes may drive CRC progression. Given the complexity of this process, pinpointing glycosylation sites directly related to CRC pathology remains a significant challenge. The above results indicate that the metric we established in this study not only provides a quantitative method to decipher the intricate N‐glycan modifications in CRC but also hints at its potential as a novel biomarker and a potential therapeutic target. This finding paves the way for innovative approaches in early CRC diagnosis and personalized treatment strategies.

Molecular markers are essential for the early detection of tumors, as indicators of clinical treatment response, and for developing personalized treatment plans. In this study, we extensively screened various IGPs and N‐glycoproteins using high‐throughput data and an optimized algorithm to identify potential CRC markers. A composite model using two glycoproteins, CLCA1 and OLFM4, was constructed with a random forest algorithm and validated in an independent public data cohort (AUC = 1). Further, IHC experiments confirmed the direct association between these proteins and CRC prognosis.

Glycosylation modifications can significantly impact protein functions and their roles in tumors.^[^ ^25^ ^]^ This study reveals the crucial role of N‐glycosylation at the APMAP‐N196 in CRC progression. We found that APMAP.N196.4200 is significantly reduced in tumor tissues and promotes CRC cell proliferation and migration in vitro. However, the specific molecular mechanisms by which APMAP‐N196 glycosylation affects CRC progression remain to be fully understood. Our findings provide an important basis and knowledge framework for future in‐depth exploration of its functions and how targeted validation of individual glycosylation sites can yield critical mechanistic insights into CRC progression.

This study identified the potential biomarkers and proposed possible mechanisms through N‐glycoproteomics analysis and machine learning algorithms. However, the limited sample size, covering only 45 CRC tumors and NATs, may restrict the generalizability of the findings and the comprehensive understanding of N‐glycosylation dynamics and biomarker validation. Moreover, given the intricate nature of glycosylation pathways, future studies should integrate additional analytical methods, such as mass spectrometry‐based N‐glycomics,^[^ ^48^ ^]^ to provide a more comprehensive understanding of N‐glycan structures and their functional implications in CRC. Future research should also validate these biomarkers in larger cohorts to assess their stability and specificity and explore their expression levels in circulating blood to evaluate their potential as liquid biopsy markers, thereby providing more practical tools for the early diagnosis, treatment monitoring, and prognostic assessment of CRC.

Overall, this study provides a comprehensive glycoproteomics map of CRC, revealing critical insights into the role of glycosylation in tumor progression and highlighting potential biomarkers for early diagnosis and therapeutic targets. Additionally, it establishes a novel framework for understanding the dynamic changes in glycosylation, potentially leading to more effective personalized treatment strategies for CRC patients.

## 4 Experimental Section

### Sample Preparation

The procedures of this study were approved by the Huashan Hospital Institutional Review Board and the approved number of the ethics committee was KY2021‐462. All patients signed the informed consent before sample collection. In total, 45 CRC tumors and adjacent tissue samples were surgically resected and collected. All samples were evaluated histologically and the identities were masked. Clinical information, including gender, age, and TNM stage, was collected (Appendix, Datasets S1, Supporting Information). Briefly, the samples were immediately washed with 1× Phosphate‐buffered saline buffer after resection to exclude the interference of high‐abundance proteins in the blood. Then, the tissues were crushed using a precooled freeze crusher and the samples were frozen in liquid nitrogen for storage before use. Technical replicates were not implemented.

### Proteins Extraction

RIPA lysis buffer (fourfold volume of tissue with protease inhibitor cocktail) was added to the clinical samples for 30 min on ice. After sonication using a contact ultrasonic crusher (15 W, 3 min) and centrifugation using a refrigerated centrifuge (16 000 × g, 4 °C, 10 min), the supernatant was collected. The protein concentration was determined by the BCA (Bicinchoninic acid) method.

### Mass Spectrometric Sample Preparation

Proteins (500 µg) were precipitated with 10% TCA on ice for 30 min and washed with pre‐cold acetone. The pellet was then mixed thoroughly in 50 mm NH_4_HCO_3_ buffer (pH 7.5). Then, the proteins were digested with sequencing‐grade modified Trypsin (enzyme to protein ratio 1:50, w/w) at 37 °C overnight. The mixed samples were reduced with 5 mm DTT for 30 min at 56 °C, followed by alkylated with 11 mm iodoacetamide for 30 min at room temperature (RT) in darkness. Samples were then acidified with trifluoroacetic acid (TFA) and desalted. 200 µg of the desalted peptides underwent enrichment utilizing a HILIC‐based enrichment procedure.^[^ ^49^ ^]^ The enriched N‐glycopeptides were desalted using a C18 column and evaporated to dryness with a SpeedVac.

### LC‐MS‐MS Analysis

Sample analysis was carried out on an EASY‐nLC 1200 UHPLC system (ThermoFisher Scientific) coupled to a Q Exactive HF‐X mass spectrometer (ThermoFisher Scientific). For proteomic analysis, 2 µg of peptides were dissolved in solvent A (0.1% formic acid) and separated via a homemade packed capillary C18 column (75 µm ID × 25 cm length, 1.9 µm particle size, Dr. Maisch GmbH) in a 120 min gradient from 6% to 90% solvent B (80% acetonitrile in 0.1% formic acid). Full mass scans were acquired with 350–1200 m/z at a mass resolution of 60 000. Ions with 2+, 3+, and 4+ charges were selected for MS/MS analysis. The 12 most intensive ions were fragmented with 28% normalized collision energy and tandem mass spectra were acquired with a mass resolution of 15000. Dynamic exclusion was set to 30.0 s and the fragments were analyzed using the HCD mode.

For N‐glycosylation omics data analysis, the N‐glycopeptides were separated with a gradient of 2 to 80% buffer B (80% CAN + 0.1% FA) within 120 min. Full scan MS spectra (mass range from m/z 700 to 1800) were acquired in the Orbitrap with a resolution of 120 000. The top 15 precursor ions were selected from each MS full scan. Subsequently, MS/MS spectra were acquired in the Orbitrap with a resolution of 15000. The AGC was 2E5 and the maximum injection time was 200 ms.

### Database Search

For proteomic data resolution, mass spectrometry data were analyzed by MaxQuant (v.1.6.15.0) using the UniProt human protein database (20376 entries, https://www.uniprot.org). Tryspin/P was specified as a cleavage enzyme, the largest missed cleavages allowed up to 2. Cysteine carbonylation was set as a fixed modification, and methionine oxidation and protein N‐terminal acetylation were designated as variable modifications.

For N‐glycoproteomic data analysis, pGlyco2.0 was used to search for original raw files enriched for N‐glycopeptides. Parameters were set to a mass tolerance of ±5 and ±20 ppm for precursor and fragment ions, respectively, the maximum missed cut site for protein digestion was 2, and cysteine carbonylation was set as a fixed modification. Alternative modifications included methionine oxidation (+15.99 Da), deamidation of asparagine (+0.98 Da), and N‐terminal acetylation of protein (+42.01 Da). N‐glycosylation (N‐X‐S/T/C; X≠P) was modified from “N” to “J”. The matching FDR of the N‐glycopeptide spectrum was set to 1% in the quality control method for intact N‐glycopeptide identification.

### Quantification of Proteome and N‐glycoproteomic

The raw levels of N‐glycopeptides were calculated by matching between run algorithms embedded in pGlycoQuant v202211 the downstream software of pGlyco2.0 according to the default parameter. To eliminate variations in N‐glycopeptide levels caused by dynamic changes in protein levels, the abundance of each identified N‐glycopeptide was normalized to the corresponding protein abundance. The homogeneity of variance between groups was calculated using the Levene test for log_2_ transformed abundance. The remove batch effect algorithm of Limma (v.3.52.1) was used for batch correction before further analysis of N‐glycosylomics and the public proteomics data due to the number of operation steps.

### Quality Control

Hela cell lysate was measured at fixed intervals as the quality‐control standard. The standard sample was digested and analyzed using the same methods and conditions as the tissue samples. To obtain reliable data, proteins or N‐glycopeptides that were present in more than 30% of the patients in the omics data were selected first. The boxplot function embedded in R and the pheatmap software package (v.1.0.12) was used for the omics data to observe differences between samples in each column. The R language Rtsne package (v.0.17), umap package (v.0.2.10.0), and prcomp package (v.0.5.1) were used for dimensionality reduction cluster analysis. When performing PCA using the prcomp function in R, all possible principal components (equal to the number of variables in the dataset) were computed by default. Additionally, the factoextra (v.1.0.7) package was used in R to generate scree plots to evaluate the contribution of each principal component. The smallest proteins or N‐glycopeptides intensity in each sample was used for missing value replications before application in all analyses unless otherwise noted.

### Difference Analysis

The leveneTest function of the R language car package (v.3.1‐2) and the t.test function of the R language were used to analyze the variance and significance of the above‐obtained proteins or N‐glycopeptides. The median between the different groups was used for comparison to obtain the fold change. The significant change was defined as a *P*‐value less than 0.05 and an absolute value of Fold Change greater than 2.

### Correlation Analysis

To compare the correlation between the proteomics data and the public proteomics data, in addition to the correlation between the proteomics data and the transcriptome data, the Cancer Genome Atlas (TCGA) RNA‐seq data was downloaded from the National Cancer Institute Genomic Data Commons by TCGAbiolinks package (v.2.26.0). The differences between Cancers and Normal tissues were normalization and tested through edgeR package (v.3.40.2). Pearson was used to calculate the gene expression similarity of different omics.

### Motif Analysis

R language was used to collate the peptide sequences corresponding to different N‐glycan forms. The protein sequences were obtained from the UniProt human protein database, and the 7 amino acids around modification sites were supplemented and truncated. The data were further analyzed by iceLogo software (v.1.0).

### Glycoform Classification

According to the monosaccharide composition with N‐linked glycans appraisal of IGP, five classes glycoforms were defined and investigated in this study: sialylated IGP (Sia, A), fucosylated IGP (Fuc, F), high mannose which represents high Hexoses (Hm, H) and high HexNAc (Hn, N), in which A glycoforms represent any N‐glycans containing sialic acid, F glycoforms represent any identified N‐glycan in recognition of IGPs containing fucose, FA glycoforms represent any N‐glycans containing sialic acid and fucose at the same time, H glycoforms represent N‐glycan in recognition of IGPs including two N‐acetylhexosamines and the others were hexoses, and N glycoforms include all N‐glycoform that do not fall into any of the above four glycoforms.

### Functional Enrichment Analysis

Cellular pathway enrichment analysis was performed based on the Gene Ontology and Reactome pathway database, and hypergeometric testing was performed in the R clusterProfiler package (v.4.6.2). Protein‐protein interaction network of dynamic proteins was established based on the STRING database and plotted in Cytoscape (v.3.10.1). The StringAPP plugin embedded in Cytoscape was used to perform functional enrichment analysis of the key network of protein‐protein interaction network. Gene set enrichment analysis (GSEA) was performed using the R clusterProfiler package and Reactome package (v.1.42.0). Gene set variation analysis (GSVA) was performed using the R GSVA package (v.1.46.0). To avoid bias, enrichment results were normalized per sample for data presentation via the pheatmap package. The variation analysis of channel information was set in the gsea‐msigdb website (http://www.gsea‐msigdb.org/gsea/index.jsp).

### N‐Glycan‐Site‐Protein‐Signaling Pathway Network

The relationship between proteins and N‐glycopeptides was sorted out using R (v.4.2.0). According to whether the log_2_ fold change was greater than 0 in more than 66.6% of N‐glycopeptides in each protein, all glycoproteins was divided into three groups: up‐regulated, down‐regulated, and no significant change. Each regulated network was classified into two categories according to the difference in the amount of high mannose or sialic acid modifications in each protein. The results were imported into Cytoscape software to draw the network, in which the color and style of nodes and edges were selected according to the interest. The results were exported to Adobe Illustrator 2024 (AI) software to add background colors for different classifications.

### ROC Analysis

The Receiver Operating Characteristic (ROC) Curve was generated using the pROC package (v.1.18.5) and the area under the curve was calculated with 95% confidence intervals. The discrimination performance of glycoprotein, glycopeptide, or model was evaluated by combining the Youden index, accuracy, specificity, and sensitivity. Random forest (v.4.7‐1.1) was used to construct a random forest classifier for eight candidate biomarkers, and the Gini importance measure was used to rank, which reflects the contribution degree of individual markers to classifier performance. The top three secreted glycoproteins with large contributions were selected after ranking according to contribution. The glm function in glmnet package (v.4.1‐8) was used to construct a multi‐indicator biomarker model for the eight candidates. To verify the reliability of the polysaccharide‐protein index, the proteomics data of CRC was obtained in the public database PDC000116 and randomly assigned the training set and test set according to the ratio of 7:3 by the strata function of sampling (v2.10).

### Cell Culture

HCT116 and HEK293T cells were purchased from the National Collection of Authenticated Cell Cultures (China) and maintained in Dulecco's modified eagle's medium supplemented with 10% (v/v) fetal bovine serum (FBS) and 1% Penicillin‐Streptomycin (P/S), at 37 °C in a 5% CO_2_ incubator. Subsequent experimental conditions depended on the applications as described where relevant in the manuscript.

### Western Blot Analysis

HCT116 cells were cultured in a complete DMEM medium with 10% FBS supplemented. The cells were harvested at 90% density and lysed with 1% SDS lysis buffer containing protease inhibitors (1 µM Pepstatin, 2.1 µM Leupeptin, 0.57 mm PMSF, purchased from Sangon Biotech). The samples were sonicated before centrifugation (16 000 × g) at 4 °C for 10 min. The protein concentration was determined by a bicinchoninic acid kit (P0010, Beyotime Biotechnology). The lysate equivalent of 20 µg of proteins was separated by gel electrophoresis using 12% sodium dodecyl sulfate‐polyacrylamide gel electrophoresis (SDS‐PAGE) and then transferred to a polyvinylidene fluoride (PVDF) membrane (10600023, General Electric Company) for protein blotting. Non‐specific binding sites were blocked in 5% Blotting Grade Blocker Nonfat Dry Milk for 1 h at RT. The membranes were incubated with primary antibodies specific for Flag (1:20 000, 20543‐1‐AP, Proteintech) or β‐Actin (1:10 000, 66009‐1‐Ig, Proteintech) at 4 °C overnight. Then washed three times in Tris‐buffered saline containing 0.1% Tween‐20 and incubated with the secondary antibodies (1:10 000, 115‐035‐003, Jackson ImmunoResearch). The bands were visualized with an enhanced chemiluminescence (ECL) kit (MAD186, Meilunbio) and recorded on Tanon 4600 (Tanon).

### Lentivirus Production, Infection, and Selection Of Infected Cells

HEK293T cells were seeded in a six‐well plate at ≈50% density 1 day before transfection. The cells were transfected using Hieff TransTM Liposomal Transfection Reagent according to the manufacturer's instructions. The mass ratio of backbone plasmid shRNA vector GIPZ targeting APMAP (used for long‐term APMAP gene silencing, shRNA sequence: tgggcttgtctttctgttt) or rescue vector UNI‐PCDH‐FLAG‐Hygro‐5 (used for long‐term APMAP gene rescuing), packaging plasmid psPAX2 and envelope plasmid pMD2.G was 4:3:3. The medium was changed 12 h post‐transfection, and the culture medium were harvested at 48 and 72 h after transfection, then filtered through a 0.22 µm filter for the following infection.

For lentivirus infection, 1 mL of DMEM complete medium was added to each well of the six‐well plate, followed by 1 mL of concentrated lentiviral particles for gene knockdown, the medium was replaced after 12 h. 48 h post‐infection, and puromycin was used to kill non‐infected cells and only resistant clones were selected and cultured. When almost all the cells in the control group died, the screening process was finished, showing that effective KD clones were obtained. Based on the knockdown stable transfected strain, the rescue cell line was constructed following the same steps as above but with hygromycin for selection.

### Wound Healing Assay

HCT116 cells rescued with Flag‐APMAP‐WT or N196D mutant were plated into 6‐well plates at a density that allowed to reach cell monolayers after 24 h. Wounds were scratched onto the monolayer of cells with a 200 µL pipette tip, and the cells were washed three times with PBS to remove suspension cells. Then the cells were cultured in DMEM medium with 1% FBS at 37 °C, 5% CO_2,_ and the cell images were captured at 0 h and 48 h. ImageJ was used to measure the wound healing rate. All assays were replicated at least three times.

### Cell Proliferation Assay

HCT116 cells rescued with Flag‐APMAP‐WT or N196D mutant were seeded in 96‐well plates at a density of 4 × 10^3^ cells per well in 100 µL of medium and cultured at 37 °C, 5% CO_2_ for 24, 48, 72, and 96 h, respectively. After that 10 µL of Cell Counting Kit‐8 (CCK‐8) reagent was added to each well and incubated for 1 h. Then the absorbance (A) of each well at 450 nm was measured by a SpectraMax M5e Microplate Reader (Molecular Devices, San Jose, CA, USA).

### Cells Transfection

Flag‐APMAP or Flag‐APMAP‐N196D plasmids were transfected into HCT116 cells using Hieff TransTM Liposomal Transfection Reagent according to the manufacturer's instructions. After 24 h, the cells were digested with trypsin and resuspended and a total of 4 × 10^3^ cells per well were seeded into 96‐well plates to perform cell proliferation assay.

### Human Tissue Microarray Analysis

IHC staining of OLFM4 (1:1000, 28432‐1‐AP, Proteintech) and CLCA1 (1:100, A15041, Abcam) were performed on paraffin‐embedded human colorectal cancer tissue microarray (HColA180Su21, Shanghai Outdo Biotech Co. Ltd., Shanghai, China), as previously described.^[^ ^50^ ^]^ The expression of each protein was quantified by three random fields of each patient using IHC scores multiplying the intensity scores with the extent of staining scores (one to nine scores) by pathologists blinded to the outcome. The association between overall survival (OS) and risk scores was assessed using multivariate Cox regression analysis. Based on multivariate Cox regression, which was performed using the R survival package (v.3.2‐13), the median risk score was used as a boundary for dividing patients into high‐ and low‐risk groups.

### Statistical Analysis

All statistical analyses were performed using R software (v.4.2.0). The relationship between two categorical variables was estimated with the *χ* ^2^ test. The relationship between a categorical variable and a quantitative variable was estimated with the Wilcoxon rank‐sum (two categories) or Kruskal‐Wallis tests (three or more categories). The relationship between two quantitative variables was estimated with Pearson's or Spearman's correlation coefficients as indicated. When appropriate, *P* values were corrected for multiple hypothesis testing with the Benjamini‐Hochberg method, or generated by permutation test to obtain a robust estimate, as specified in the text or figure legends. Survival was analyzed with Kaplan‐Meier survival estimates and log‐rank tests, which were performed using the R survival package (v.3.2‐13) and survminer package (v.0.4.9). All tests were two‐sided if not specifically indicated. No statistical methods were used to predetermine the sample size. The experiments were not randomized, and investigators were not blinded to allocation during experiments and outcome assessment unless stated otherwise.

## Conflict of Interest

The authors declare no conflict of interest.

## Author Contributions

G.L. and L.C. contributed equally to this work. H.H., Q.L., and Q.M. conceived the research and designed the studies. G.L. performed bioinformatics analyses and visualization and wrote the manuscript. L.C., Y.G., and X.M. were involved in clinical sample collection and clinically relevant research under the supervision of Q.L. and Q.M. J.Z. performed the biological experiments, and were involved in the manuscript writing. X.R. and Y.J. participated in the analysis of proteomics samples. K.L. contributed to data analysis. H.H. supervised the study and revised the manuscript. All authors have approved the final version.

## Supporting information



Supporting Information

Supporting Information

Supporting Information

Supporting Information

Supporting Information

Supporting Information

## Data Availability

The mass spectrometry data have been deposited to the ProteomeXchange Consortium via the PRIDE partner repository with the dataset identifiers PXD055935 and PXD055957.
