# Supplementary material for: Deciphering the Metabolic Impact and Clinical Relevance of N‐Glycosylation in Colorectal Cancer through Comprehensive Glycoproteomic Profiling
Source: Adv Sci (Weinh). 2025 Apr 26;12(21):2415645. doi: 10.1002/advs.202415645 (PMC12140321; doi:10.1002/advs.202415645)
Supplement: Supplementary file 1 — Supporting Information [file ADVS-12-2415645-s005.docx]

Supporting Information

Deciphering the Metabolic Impact and Clinical Relevance of N-Glycosylation in Colorectal Cancer Through Comprehensive Glycoproteomic Profiling

Guobin Liu, Lu Chen, Jingxiang Zhao, Yue Jiang, Yarong Guo, Xiang Mao, Xuelian Ren, Kun Liu, Qi Mei*, Qunyi Li*, He Huang*

**Includes**:

Figures S1 to S6

Legends for Datasets S1 to S4

Other supporting materials for this manuscript include the following:

Datasets S1 to S4


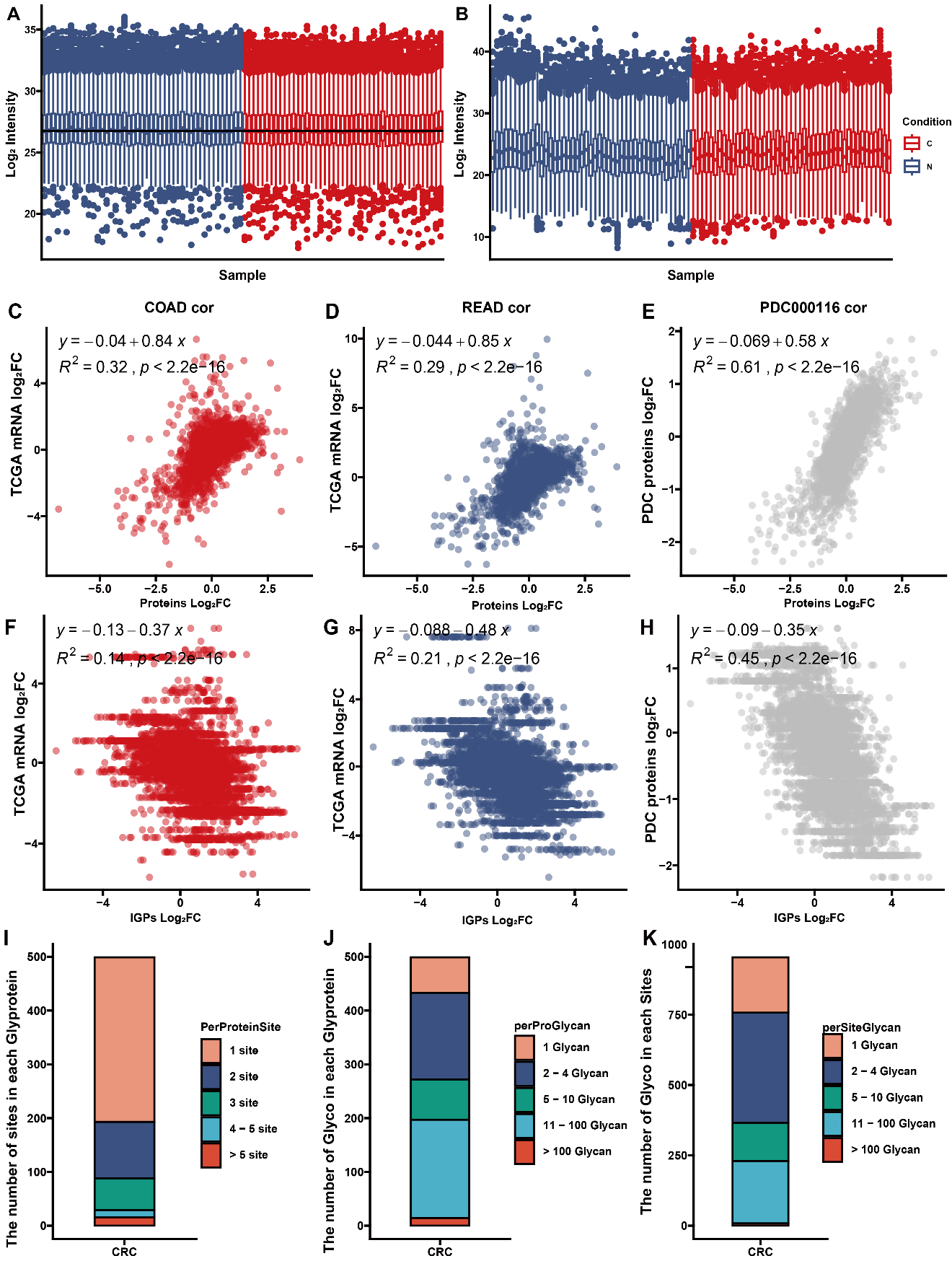


**Figure S1.** Quality control measurements of global proteomics and N-glycoproteomics. (A) Normalized log_2_ protein expression ratios for all samples. Red bars indicate tumors and blue indicates NATs. (B) Normalized log_2_ IGPs expression ratios for all samples. Red bars indicate tumors and blue indicates NATs. (C) Comparative analysis of differential abundance changes in proteins (our data) and mRNAs in TCGA Colon adenocarcinoma (COAD) tumors versus NATs. (D) Comparative analysis of differential abundance changes in proteins (our data) and mRNAs in TCGA Rectum adenocarcinoma (READ) tumors versus NATs. (E) Comparative analysis of differential abundance changes between proteins from the PDC00016 label-free proteomics cohort and our data in tumors versus NATs (PDC000116). (F) Comparative analysis of differential abundance changes in IGPs from our data and their corresponding mRNAs in TCGA-COAD tumors versus NATs. (G) Comparative analysis of differential abundance changes in IGPs from our data and their corresponding mRNAs in TCGA-READ tumors versus NATs. (H) Comparative analysis of differential abundance changes in IGPs from our data and their corresponding proteins in tumors versus NATs (PDC000116). (I) Distribution of glycosylation sites per glycoprotein. Different colors represent the number of glycosylation sites per glycoprotein. (J) Distribution of N-glycans per glycoprotein. Different colors represent the number of N-glycans per glycoprotein. (K) Distribution of N-glycans per glycosylation site. Different colors represent the number of N-glycans per glycosylation site.


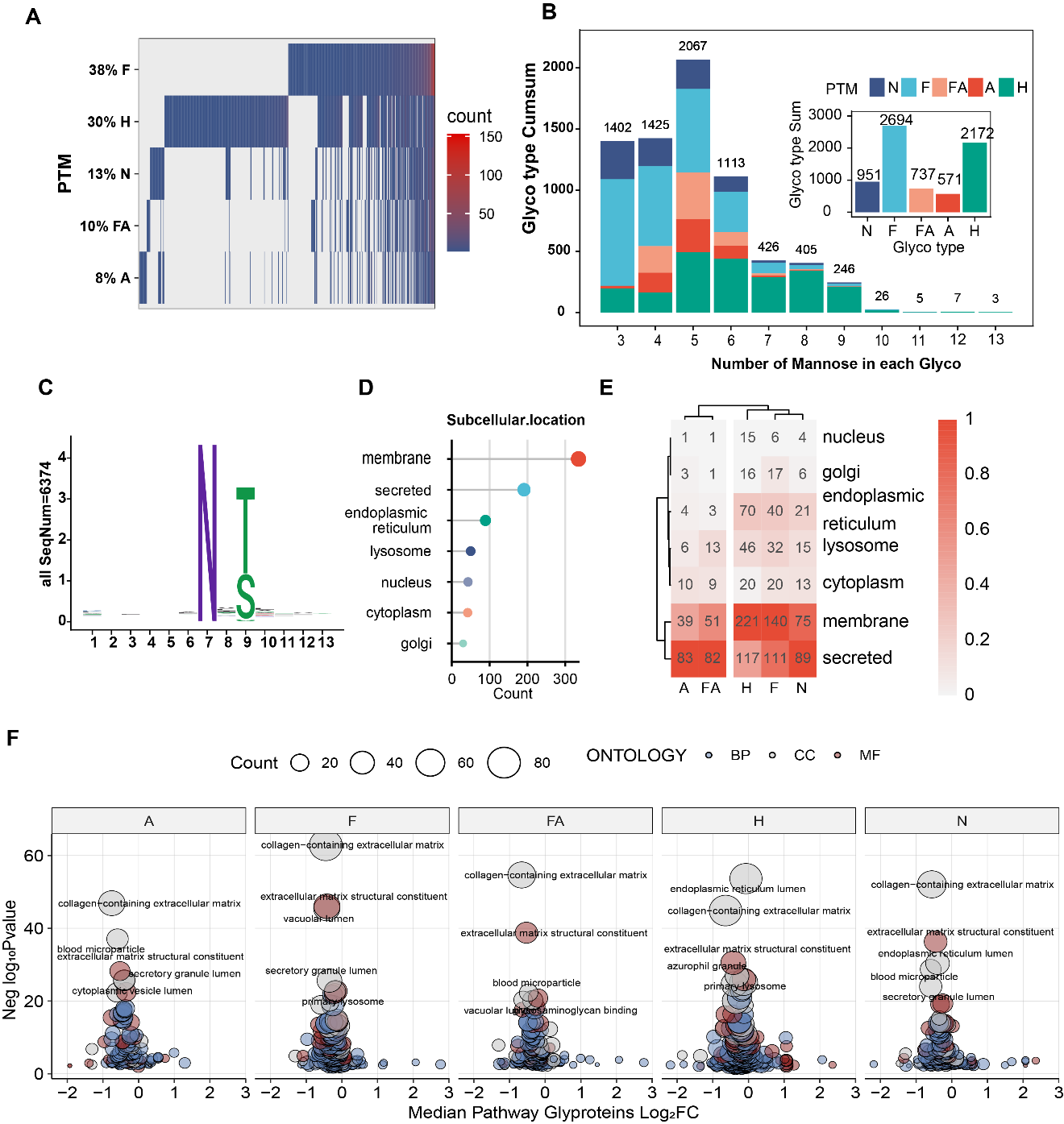


**Figure S2.** Annotation and enrichment of differential IGPs. (A) Heatmap showing the number of glycoforms per glycoprotein. Each column represents a glycoprotein, and colors indicate the count of IGP types per glycoprotein. The number on the left represents the percentage of each glycoform relative to the total. Different colors represent different glycoforms. (B) Bar plot displaying the monosaccharide composition of each IGP, according to the amount of mannose. (C) Motif analysis of all identified IGPs. (D) UniProt subcellular localization summary for all IGP proteins. Different colors represent different subcellular localization. (E) UniProt subcellular localization summary for each individual glycoform of glycoproteins. (F) GO pathways enrichment analysis for each glycoform of glycoproteins. Different colors represent different pathways and size represent the scale P-value.


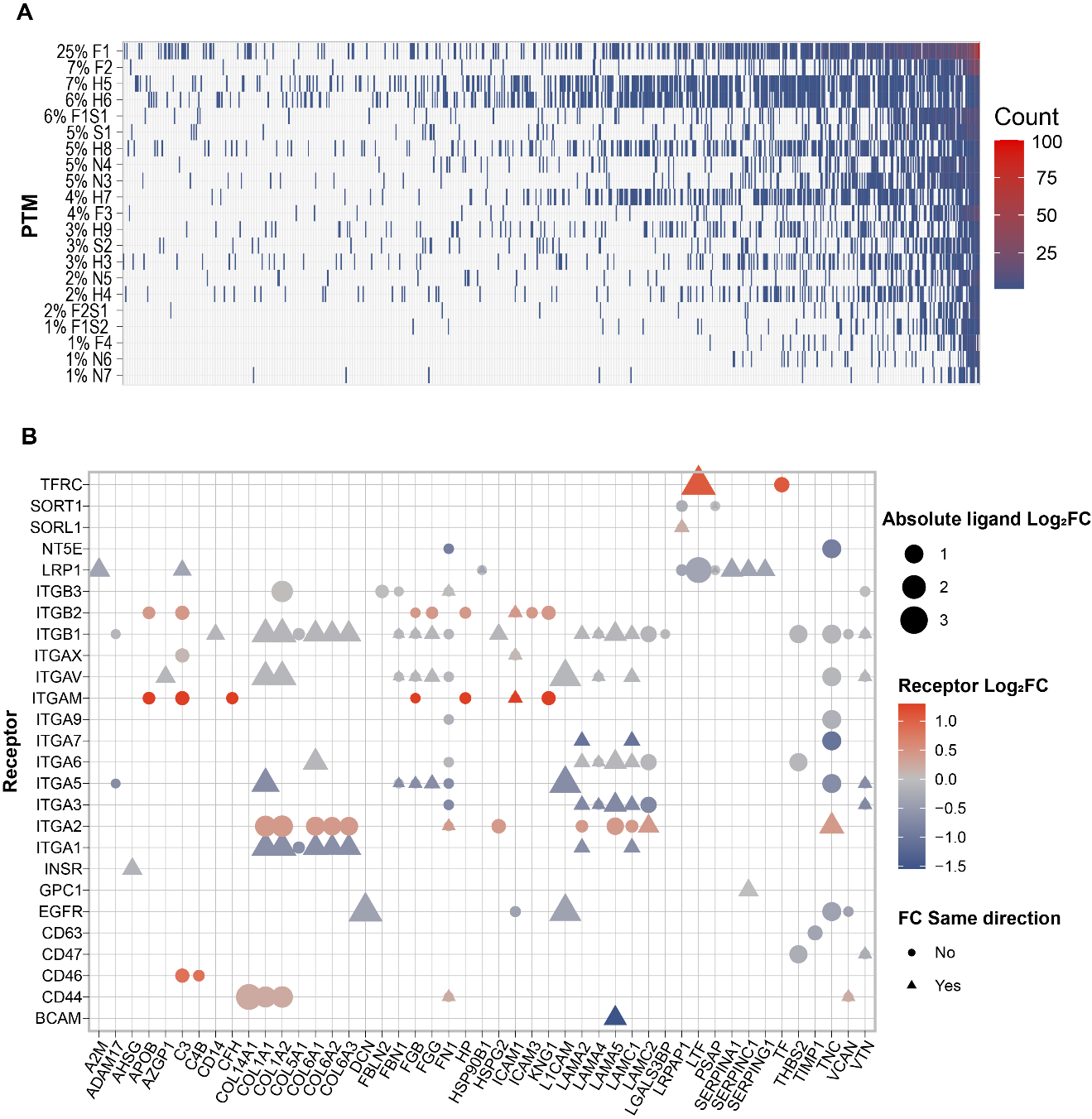


**Figure S3.** Comparative analysis of differential abundance changes in receptor and ligand proteins. (A) Heatmap displaying the number of subdivided glycoforms per glycoprotein. Each column represents a subdivided glycoprotein, and colors indicate the count of IGP types. The left number shows the percentage of each subdivided glycoform relative to the total. (B) Comparative analysis of differential abundance changes in receptor and ligand proteins, annotated via the celltalker package. Dot colors represent the log_2_ fold change in N-glycosylated receptors, dot size indicates the absolute log_2_ fold change in N-glycosylated ligands, and dot shape represents the synchronous expression between the ligand and receptor.


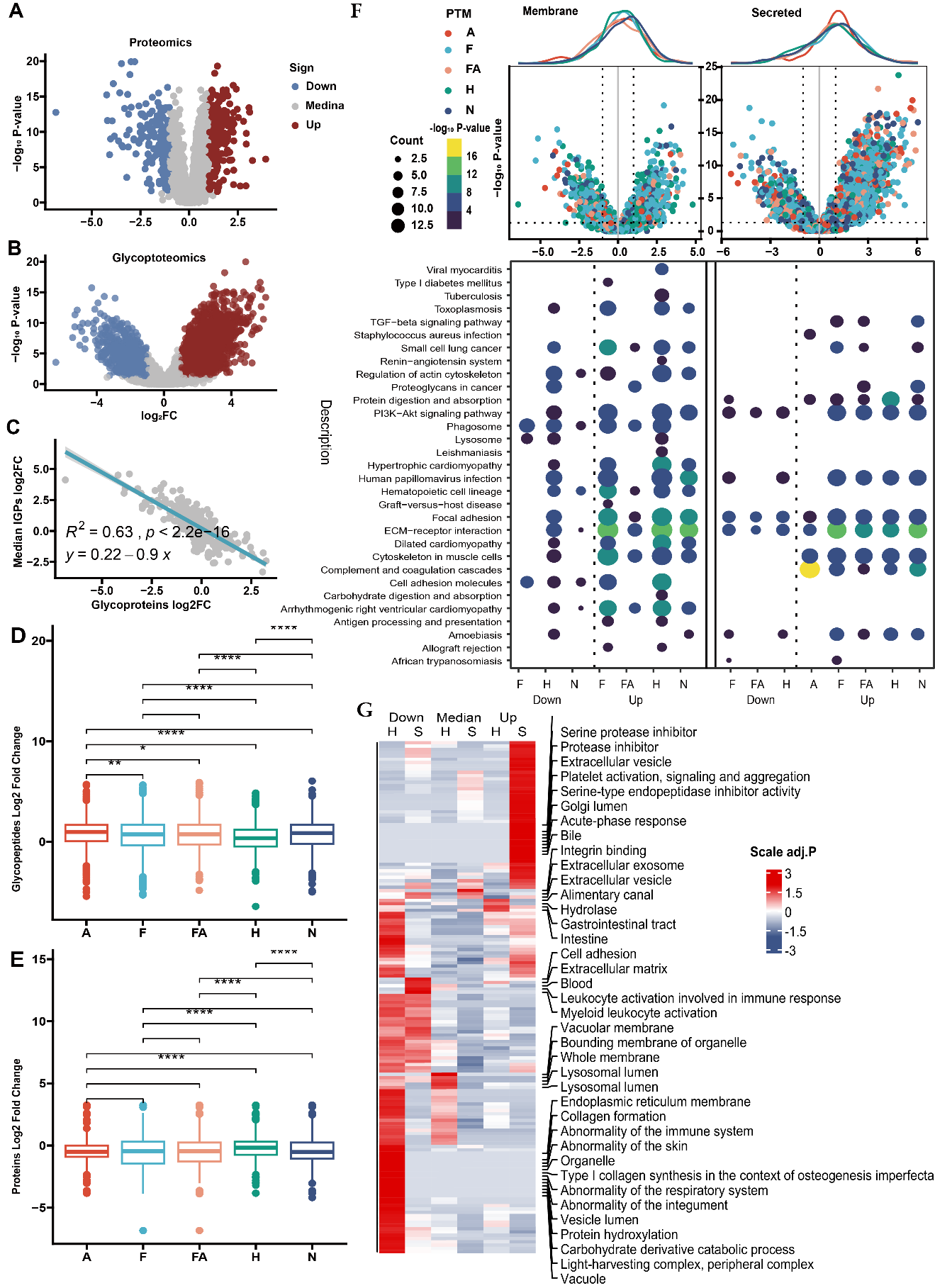


**Figure S4.** Functional analysis of glycosylation with different expression patterns. (A) Volcano plot of differentially expressed IGPs in tumors versus NATs. Red dots indicate significantly upregulated proteins (log2FC > 1, adjusted P-value < 0.05), blue dots represent significantly downregulated proteins (log2FC < -1, adjusted P-value < 0.05), and gray dots denote proteins without significant changes. (B) Volcano plot of differentially expressed proteins in tumors versus NATs. Red dots indicate significantly upregulated proteins (log2FC > 1, adjusted P-value < 0.05), blue dots represent significantly downregulated proteins (log2FC < -1, adjusted P-value < 0.05), and gray dots denote proteins without significant changes.(C) Comparative analysis of the median differential abundance of IGPs and their corresponding glycoproteins. (D) Boxplot showing the log_2_ fold change of IGPs from each glycoforms (P-value from Wilcoxon signed-rank test). Different colors represent different glycoforms. (E) Boxplot showing the log_2_ fold change of glycoproteins corresponding to each glycoforms (P-value from Wilcoxon signed-rank test). Different colors represent different glycoforms. (F) Volcano plot showing differentially expressed membrane-located or secreted glycoproteins of IGPs in tumors versus NATs and different colors represent different glycoforms. The bottom part of the figure displays the corresponding Gene Ontology (GO) pathway enrichment results and different colors represent the scale P-value. (G) Heatmap displaying GO pathway enrichment result for six groups.


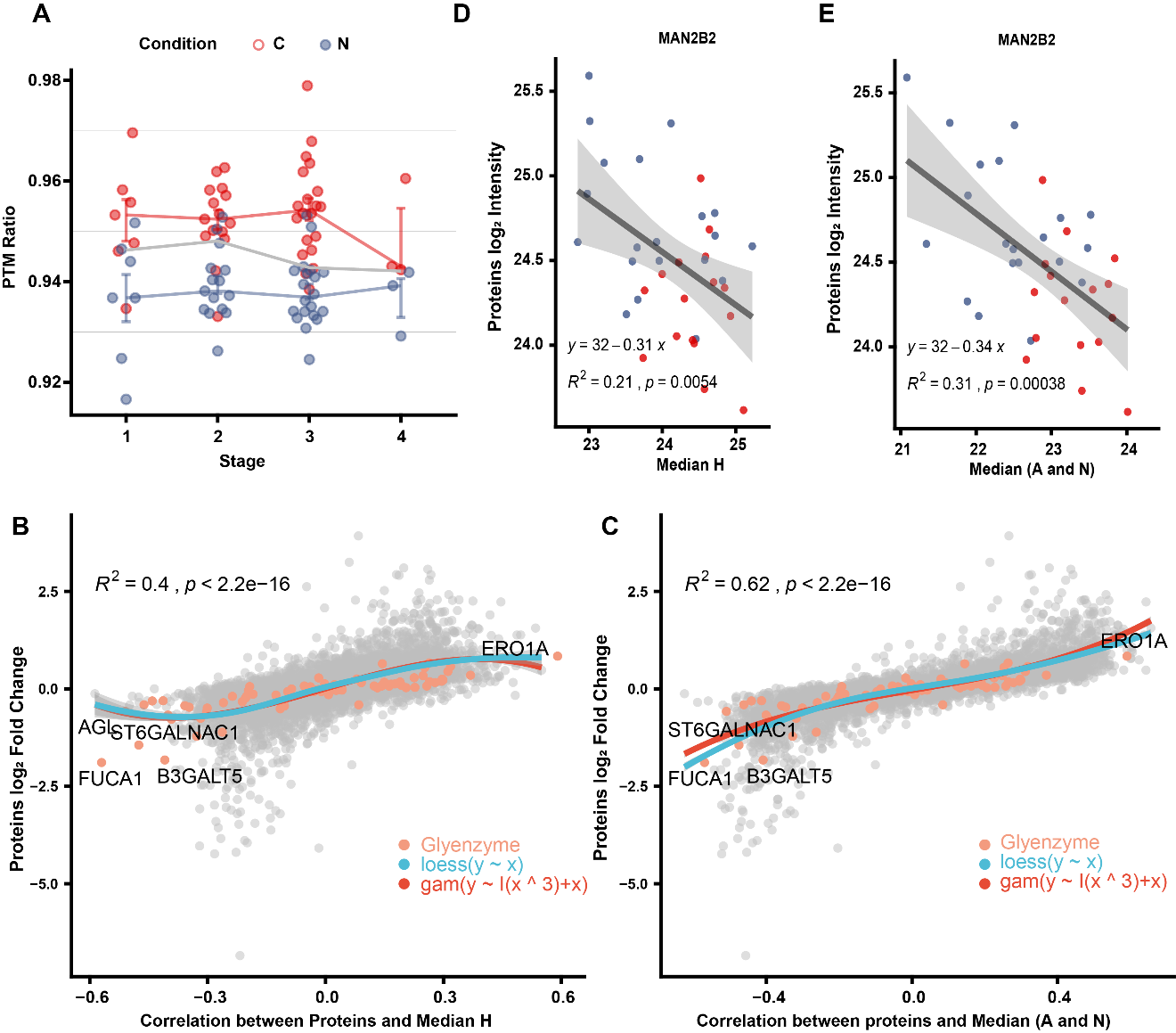


**Figure S5.** Identification for robust glycoform scores associated with CRC development (related to Figure 4). (A) Plot showing optimal combination results for each stage tissue stage. Red dots indicate tumors, and blue indicates NATs. (B) Correlation between Pearson correlation values and log_2_ median differential protein abundance. Correlation values are derived from Pearson correlation between log_2_ median H and each protein detected in our proteomics data (*P-value* from Pearson correlation). Orange color represents N-glycosylation enzymes. (C) Correlation between Pearson correlation values and log_2_ median differential protein abundance. Correlation values are derived from Pearson correlation between log_2_ median A and N and each protein detected in our proteomics data (*P-value* from Pearson correlation). Orange color represents N-glycosylation enzymes. (D) Pearson correlation between log_2_ median H and MAN2B2 log_2_ intensity abundance. Red dots indicate tumors, and blue indicates NATs. (E) Pearson correlation between log_2_ median A and N and MAN2B2 log_2_ intensity abundance. Red dots indicate tumors, and blue indicates NATs.


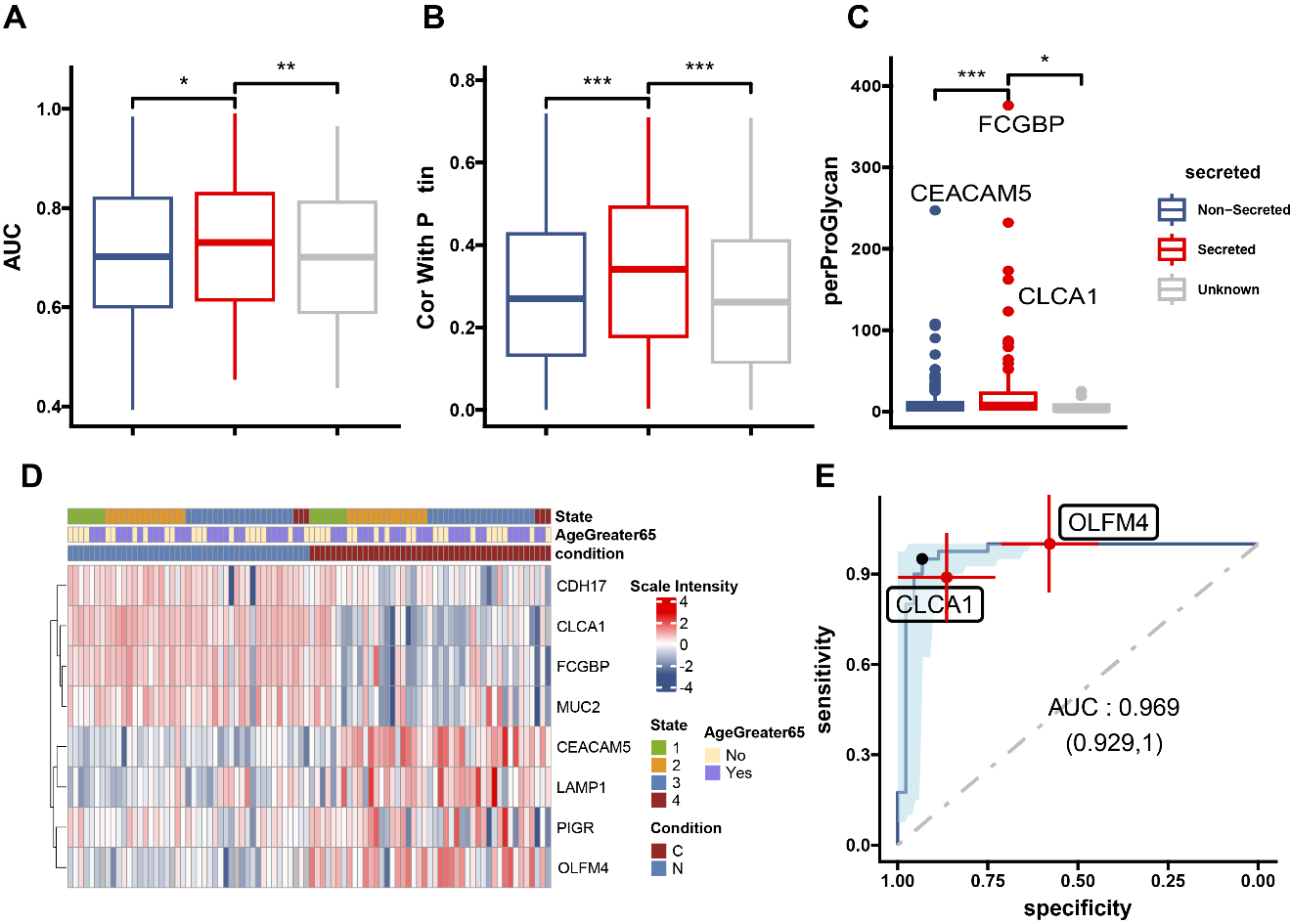


**Figure S6.** Screening potential CRC prognostic signatures (related to Figure 5). (A) Boxplot comparing the distribution of glycoprotein power to identify CRC among the three groups from UniProt annotation (*P-value* from Wilcoxon signed-rank test). Red bar indicates secreted proteins, grey indicates unknown proteins, and blue indicates non-secreted proteins. (B) Correlation with glycoforms ratio scores among the three groups from UniProt annotation (*P-value* from Wilcoxon signed-rank test). Red bar indicates secreted proteins, grey indicates unknown proteins, and blue indicates non-secreted proteins. (C) Boxplot comparing the distribution of IGP counts between the three groups (*P-value* from Wilcoxon signed-rank test). Red bar indicates secreted proteins, grey indicates unknown proteins, and blue indicates non-secreted proteins. (D) Heatmap displaying the expression of 8 glycoproteins across all samples. (E) ROC curve showing the logistic regression models of Mutule glycoproteins for CRC identification. Each red dot represents an independent ROC value for each protein.

**Table** **S1**. Clinical data from 45 CRC patients.

**Table** **S2.** Proteomics data of 45 tumors versus NATs.

**Table** **S3.** Glycoproteomics data of 45 tumors versus NATs.

**Table** **S4.** Clinical data from 87 CRC patients, including immunoblot scores for OLFM4 and CLCA1.
